# Planning policy, sustainability and housebuilder practices: The move into (and out of?) the redevelopment of previously developed land

**DOI:** 10.1016/j.progress.2012.10.001

**Published:** 2013-05

**Authors:** Nikos Karadimitriou

**Affiliations:** University College London, Bartlett School of Planning, Wates House, 22 Gordon Street, WC1H 0QB, United Kingdom

**Keywords:** Housebuilding, Planning policy, Urban policy, Business strategy, Urban regeneration

## Abstract

This paper explores the transformations of the housebuilding industry under the policy requirement to build on previously developed land (PDL). This requirement was a key lever in promoting the sustainable urban development agenda of UK governments from the early 1990s to 2010 and has survived albeit somewhat relaxed and permutated in the latest National Planning Policy Framework (NPPF). The paper therefore looks at the way in which the policy push towards densification and mixed use affected housebuilders’ business strategy and practices and their ability to cope with the 2007 downturn of the housing market and its aftermath. It also points out the eventual feedback of some of these practices into planning policy.

Following the gradual shift of British urban policy focus towards sustainability which started in the early 1990s, new configurations of actors, new skills, strategies and approaches to managing risk emerged in property development and housebuilding. There were at least two ways in which housebuilders could have responded to the requirements of developing long term mixed use high density projects on PDL. One way was to develop new products and to employ practices and combinations of practices involving phasing, a flexible approach to planning applications and innovative production methods. Alternatively, they could approach PDL development as a temporary turn of policy or view mixed use high density schemes as a niche market to be explored without drastically overhauling the business model of the entire firm. These transformations of the UK housebuilding sector were unfolding during a long period of buoyancy in the housing market which came to an end in 2007. Very little is known both about how housebuilder strategies and production practices evolved during the boom years as well as about how these firms coped with the effects of the 2007 market downturn.

The paper draws on published data (company annual reports, government statistics) and primary material (stakeholder interviews, planning applications, unpublished project specific information) to explore two different approaches that two major housebuilders (the Berkeley Group and George Wimpey – now Taylor Wimpey) followed during the boom years in response to the changing requirements, risks and uncertainties embedded in the residential development process. The recent turmoil in the property markets acted as an ‘acid test’ to business models and practices and not all firms survived it. What is more, the UK government is now embedding some of those business practices into policy, thus completing one loop in a co-evolving feedback spiral between planning policy and business strategy.

## Highlights

► Shifts in urban policy linked to new business models, strategies and skills in housebuilding. ► Housebuilding output becoming polarised in terms of dwelling type and geographical allocation. ► London market ‘cornered’ by Berkeley, focusing on high end regeneration schemes. ► Wimpey adversely affected by the downturn, now recovering following the Taylor Woodrow merger. ► Community consultation a key future challenge for housebuilders.

## 1 Introduction

The emergence of the sustainability agenda during the last three decades brought to the fore considerations of balancing economic growth with environmental and social concerns. One of the effects it had on urban policy in a UK context was a renewed attempt to bring development back into the cities, to promote compaction, mixed uses, urban living and sustainable communities. Almost 18 years ago, in 1995, the introduction of quantitative targets for the percentage of new dwellings to be built on previously developed land (PDL) marked the launch of a series of policy initiatives that affected the business environment in which the housebuilding sector is currently operating. Thus, the redevelopment of PDL became a major consideration for everyone engaged with the way the built environment is produced, consumed and experienced. In spite of the decision by the UK government to remove the quantitative policy targets attached to PDL redevelopment, the inextricable links between the sustainability agenda and land redevelopment means that the issue should remain topical, one way or another, for the foreseeable future. What is more, measures like the introduction of the pre-application consultation requirement as part of the Localism Act (UK Government, 2011) are reinforcing the diffusion amongst developers of business practices originally implemented by housebuilders who wanted to address the risks of large scale mixed use PDL redevelopment schemes.

The long term implications of the policy shifts described above should not be underestimated. The requirements for PDL redevelopment marked a turning of the policy tide away from ‘anti-urbanism’/‘decentrism’ towards ‘urbanism’/‘centrism’ and city ‘compaction’ (Breheny, 1996) and thus the beginning of an era of government policy promoting urban settlements with a wide social and land use mix in an effort to combine environmental, social and economic goals. Documents like the Urban Task Force (UTF) report (Urban Task Force, 1999) or the “Planning for the Communities of the Future” White Paper (DETR, 1998b) reflected the spirit of that time and proved very influential in setting the agenda and in structuring future debates and policy directions (see DETR, 2000a). The cautious reactions of several stakeholders to the effective abolition of the sequential approach and the ‘brownfield test’ in an interim version of the National Planning Policy Framework (NPPF) (DCLG, 2011) and the more positive reception of their restitution in a later version (DCLG, 2012) demonstrates how deeply embedded in policy discourse the densification approach has now become as part of the polysemantic sustainability discourse.

As will be examined in the following sections of this paper, this consistent policy turn, in part expressed through the promotion of land recycling and in part expressed through the densification and mixity imperatives, had significant consequences for the production of the built environment and even more so for housing production in the UK. It was within a context of rampant house price inflation that housebuilders had to address this fundamental shift of their business environment. In rather broadbrush terms, so far as housebuilders were concerned, this policy shift meant amongst other things that the sources and the types of land available to them were to be dramatically altered. Given the importance of land inputs to any housebuilder's business model it would not be an exaggeration to call this change in their business environment an instance with potentially significant effects on their survival prospects.

Evidence supporting the view that “new ways of doing things” were emerging in the production of housing in the UK, affected by changes in policy and thus in the nature of land availability and customer demand was offered by Karadimitriou (2003) whereas this argument was further elaborated in Adams (2004) and Karadimitriou (2005). Following the 2007/2008 market downturn and the subsequent change in government in 2010 there is renewed scope to look into what these new ‘ways’ have developed into, how they relate to the current business and policy environment, their effects on the organisation of housebuilding firms and on housebuilder business strategies as well as how these strategies coped with the market downturn and the subsequent stabilisation at a lower price and output level.

This paper will argue that there is significant benefit to be gained in terms of our understanding of the housebuilding sector from conceptualising housebuilding companies as profit seeking organisations with a diverse range of objectives, the most crucial of those being the survival of the organisation itself. Faced with a shift in the policy environment, housebuilders developed strategies and practices that aimed at ensuring survival and profitable operation within that environment. These strategies and practices more often than not evolved from pre-existing capacities combined with newly developed skills.

Although the strategies and practices examined in this paper emerged in response to the changing policy environment, the objectives that they satisfy do not necessarily align with the objectives of policy itself. What is more, as the case of the ‘pre-application consultation’ highlights, Government policy and actor strategies are often tied in a process of constant interaction and mutual adjustment. The production of the built environment not only reflects this process of co-evolution of policy and business practices but also influences it, thus putting in place the preconditions for the next economic cycle.

In terms of methodology, this research is based on a choice of cases made with replication rather than sampling in mind. This means that each selected site and firm serves the specific purpose of being useful in producing similar results or “contrasting results but for predictable reasons” (Yin, 1992, p. 46). Berkeley is a company often referred to as one of the best run and most astute strategically, a sector leader and a trendsetter. George Wimpey was a rather typical example of a top-10 housebuilding company following a more traditionalist approach closely associated with greenfield development.

This case study research strategy has both a historical as well as a direct observation/interviewing character. The use of multiple sources of evidence allows for corroboration of the information thus increasing the validity and the accuracy of the research findings. The paper follows a combined quantitative and qualitative approach in order to analyse the primary data collected via interviews and archival research and the secondary data found in published government statistics, databases and company annual reports. Aggregate macro data published by the DCLG and relating to land use change, the housebuilding sector and housing production have been analysed mainly through the use of descriptive statistics in order to understand the shifts taking place at the level of the housebuilding sector and in the country as a whole. Firm-related financial data mainly deriving from company annual reports have also been analysed quantitatively to calculate simple ratios (margins, etc.) that paint a clearer picture of company performance.

Qualitative directed content analysis (see Hsieh & Shannon, 2005) of interviews, printed and electronic material was used in order to understand issues relating to corporate strategy and norms and routines at the project level. The Estates Gazette Interactive (EGi) property database was used in order to uncover the transaction history of each site and the actors involved with each site at key points in time. Planning application information and project information was retrieved by using the online portal of the local planning authority (Wandsworth). Articles from online editions of daily newspapers (The Daily Telegraph, The Financial Times) and specialised journals (Estates Gazette, Planning Magazine, Housebuilder Magazine) where used in order to find out more information about key points in corporate history (like mergers and acquisitions).

Altogether 12 key individuals where interviewed using semi-structured face to face extended interviews (or phone interviews in two cases where a visit was not possible). The questionnaires covered eight different areas of interest: resource inputs in the development process, actor roles and relationships, corporate strategy, corporate assumptions about and expectations for the future, established norms, rules and routines and the way they apply in the company's operations, the effects of changes in planning policy on corporate strategy and operations/project development, financial information relating to the project, site specific information and history. The interviewees included senior managers/directors, project managers, architects and planners in both companies. In addition to corporate interviewees a second set of interviews was done with local authority planners and local community representatives though it was specifically focused on site history, objections to the development and the approach that each housebuilder and the local authority followed. These interviews, together with references in the media allowed in many instances to triangulate the various statements found in the annual reports on which the research drew heavily in order to understand the views that the company's management expressed to the shareholders and the world at large.

It is not possible nor would it be desirable to claim that what the research uncovers can be extrapolated to the industry as a whole. The research design and scope does not allow it, however the research uncovers two rather important responses from two firms and shows how they could have an effect at a wider scale as they serve as plausible explanations of what other firms might be doing. It is both through statistical or other quantitative methods as well as case studies that any inferences could be made. This approach is suggested amongst others in the work of Guy and Henneberry (2002) as the way critical science should approach its object of examination. However, more research into other firms’ attitudes and strategies could shed more light into industry-wide trends.

Following the introduction, the paper discusses the evolutionary character of business strategy and practice and explores the co-evolutionary relationship of business and policy. It summarises developments in planning policy that affected the land supply regime and the requirements these changes placed on housebuilders during the last 20 or so years. Within this context, housebuilders found themselves faced with a set of challenges, some familiar to them and some others less so. Section 3 moves on to explore the macroscopic changes in the output of the housebuilding industry and their link to policy. In Section 4 the paper looks into how the strategies of two prominent housebuilders evolved first as a response to the changing policy environment and then as a result of the recent market downturn. Section 5 explores those strategies further and delves at the project level by looking into two PDL development projects, one for each developer. It therefore highlights the different norms and routines that each housebuilder developed at the project level as a result of their strategic realignment within the context of a volatile market. The last section summarises the key findings and concludes that although it cannot be claimed that the cases examined in the paper demonstrate the full range of strategies and practices one could encounter they nevertheless highlight two distinct instances of housebuilder adaptation with significantly different outcomes.

## 2 The co-evolution of planning policy and business strategy

As far as housing and planning policy are concerned, the shift to PDL re-use was primarily introduced through a stream of regularly updated policy documents mainly in the form of White papers and Planning Policy Guidance (PPG) notes. For example, following the Department of the Environment's new environmental strategy (DoE, 1990) the PPG3 of 1992 directed housing development towards more use of brownfield land. Then, following the 1995 White Paper ‘Our future homes’ (UK Government, 1995) and the concern caused by future household growth projections reflected in “Household Growth: Where Shall We Live?” (UK Government, 1996) the government set a target for 50% of new housing development to be built on brownfield land, raised to 60% in 1998, shortly after the Labour party was elected to power.

A few years later the revised PPG3 of 2000 (DETR, 2000b) introduced the housing capacity studies and the sequential approach to the release of land for development. It also required LPAs to re-examine existing land allocations to fit the PDL re-use, densification, mixed use and social inclusion policies. It was further strengthened by Planning Policy Statement (PPS) 1 of 2005 (ODPM, 2005b) which set out the key sustainability principles that would guide planning policies and decisions for years to come. The updated PPG3 (ODPM, 2005c) continued along the same lines and soon thereafter PPS3 (DCLG, 2006) and the Regional Spatial Strategies (RSS) framework modified but, if anything, enhanced this policy regime even further. Thereafter, Local Authorities were bound to have PDL ‘land targets’ and ‘trajectories’ in place, in compliance with the Regional and National policy and targets. By the end of 2008. Caroline Flint (Minister of State for Housing and Planning) could plausibly comment that:

The re-use of Brownfield land lies at the heart of a wide range of Government policies and English Partnerships’ work in developing a National Brownfield Strategy is an important step towards achieving our ambitious targets for housing growth and underpins our policy for the revival of our towns and cities and for achieving more sustainable patterns of development. (DCLG, 2008, p. 3)

Following the change of government in 2010 and the subsequent phasing out of the RSS-PPS system, the NPPF introduces a set of new policy directions and requirements with a view to facilitating economic growth. Its latest version (DCLG, 2012) quotes the re-use of PDL as one of its core principles and applies a sequential test to the location of ‘main town centre uses’.

In this sustainability discourse ‘greenfields’ and ‘brownfields’ have been counterposed in a dialectical way, as polar extremes, examples of what is good and what is not (Karadimitriou, Doak, & Cidre, 2010; Murdoch, 2004). The ‘clean’ and ‘natural’ greenfields became even more sacred. Their well-entrenched sanctity was reaffirmed through their juxtaposition against the equally ill-defined category of ‘contaminated’ and ‘derelict’ brownfields, positioned at the opposite extreme. In a society where the ‘rural idyll’ has exceptional cultural significance it should not come as a surprise that any attempt to divert the course of urban development from an expansionary paradigm towards an ‘urban renaissance’ would be accompanied by strong symbolical references to the greenfields and the brownfields.

By the same token however, brownfields have become a more acceptable option for future development to be directed into. Since the early 1990s both Conservative and Labour governments, have subscribed to this agenda and have pursued it with remarkable consistency. The planning system, through the control of the release of land for development, was used as a mechanism to promote this agenda. This in turn affected the types of built environments the development industry, and therefore housebuilders, were called upon to produce. In the current UK context this intervention through the planning system is probably one of the few ideologically acceptable ways through which government can actually intervene in order to direct private business interests.

It can be plausibly argued that the outcome of the densification policies of the last couple of decades was the disruption of the flow of developable greenfield land into housebuilders’ landbanks and the devalorisation of their existing landbanks as it became increasingly difficult to gain planning permission for developing them. Instead, PDL became a key component of total land supply for which housebuilders knew they stood a good chance of getting permission to build on. This change in policy however imposed more than a substantive economic cost to housebuilders: it required them to adjust their operation in order to be able to provide the new types of residential built environments that both policy and the economics of PDL redevelopment require. It would be unreasonable to expect the whole industry to approach this challenge from exactly the same angle, respond to it in exactly the same way and at exactly the same time. One also has to bear in mind that apart from anything else, most of the period 1995–2007 was characterised by unprecedented house price inflation that created an environment favourable to the housebuilding sector's profitability and made any need for strategic realignment much less pressing.

According to Porter (1980, 1985) technological, political, or social changes are the key factors affecting the business environment any firm is operating in, a point also made by Leonard-Barton (1998). The factors that Porter recognises as having potentially strategic importance are “long run changes in growth; changes in buyer segments served; buyers’ learning; reduction of uncertainty; diffusion of proprietary knowledge; accumulation of experience; expansion (or contraction) in scale; changes in input and currency costs; product innovation; marketing innovation; process innovation; structural change in adjacent industries; government policy change; entries and exits” (Porter, 1985, p. 164).

In his discussion of paradigm shifts, Dosi (1982) argues that ‘economic forces’ and ‘institutional and social factors’ work as filters, ‘selective devices’, that allow only certain ‘paths’ to be followed. However, the set of feasible futures constitutes a much larger pool from which the selection is made. Once a paradigm is established, exactly because of the ‘bounded’ nature of human rationality, it has a powerful ‘exclusionary’ effect in the sense that it becomes the ‘normal’ way of doing things and therefore focuses the efforts of relevant agents in ‘refining’ it rather than substituting it altogether. This refinement process is the ‘normal’ technological progress. In similar fashion to the management literature Dosi and Marengo (1993) argue that the capacity of the firm to adapt within each ‘path’ or, even more, to contribute to the ‘paradigm shift’ is determined by the “problem-solving features of particular sets of organisational interactions, norms and – to some extent – explicit strategies” in other words ‘competencies’ comprise the set of organisational routines of the firm whereas strategies are seen as the higher-level link between competencies and the external environment.

These ideas are echoed in Porter's arguments on the competitive strategies and the competitive advantage of industries (Porter, 1980, 1985, 1998). Institutional structures, such as ‘ways of doing things’ or ‘paradigms’ arise through habitual behaviour (Gruneberg & Ive, 2000a, p. 22), through repetition of routines starting from the individual but extending up to the level of the firm. Indeed one of the firm's main functions is to ensure that individuals do behave according to certain routines in order to achieve specific aims of that organisation (Nelson & Winter, 1982). In that respect, the notion of collaborative advantage deriving from either interunit collaboration (Hansen & Nohria, 2004) or collaboration between stakeholders (Huxman, 1996) will be of particular interest when this paper looks into practices affecting the competitive advantage of the two firms.

The firms of today have inherited many of their characteristics from the past. In the words of Hamel and Prahalad (1994) each firm is characterised by the “… biases, assumptions and presuppositions…” of managers, as well as “…beliefs, values and norms…” about markets, clients, stakeholder interests, competitors, etc. They interchangeably call these established sets of norms and routines ‘managerial frames’ or ‘genes’/’genetic coding’, meaning: an established social structure with its own rules and routines and self-replication dynamics. This ‘code’ is a result of a particular set of environmental conditions. When the environment changes this ‘genetic inheritance’ may actually become a threat to the firm's survival and the firm will need to shed it. Business strategy is then a way to optimise a firm's response to its environment by exploiting its strengths and its weaknesses in the face of constantly changing opportunities and threats (Montgomery & Porter, 1991).

Porter sees strategy as the process of combining activities in order to gain an advantageous position out of the many possible and asserts that the only way for a business to perform better than its rivals is to “establish a difference that it can preserve” (Porter, 1998, p. 40). He therefore concludes that “Competitive strategy is about being different. It means deliberately choosing a different set of activities to deliver a unique mix of value” (Porter, 1998, p. 45). The idea promoted by Porter (1980, 1985, 1998) is that a company's view of new ‘ways of doing things’ depends on the usefulness of this ‘new way’ in establishing difference, thus assisting the company's competitive strategy. Insofar as diffusion is concerned, if a ‘new way’ offers a competitive advantage then it is likely to find imitators who will increase in size and number, whereas the size and number of those who stick to ways unsuitable to the new environment should presumably decline. As a result, this dynamic should affect the size of output as well as the relative presence of different types of businesses. As in any such process of adaptation, the element of time is key in the sense that it often can take several years for such dynamics to play out and for the outcomes to become noticeable at the aggregate level. Although a crisis is not a necessary requirement for business practices to evolve it does put existing business models to the test. Thus, business practices (for example high gearing) that may have appeared to be suitable for the circumstances of a property boom may prove destructive during a property market downturn.

Whereas it may be easier to quantitatively examine^1^ the outcomes of a selection process, the examination of the patterns of actor behaviour requires an examination of the strategies and the learning process of organisations or individuals. Here is how Nelson and Winter (1982) summarise the basic characteristics of the evolutionary approach:

• The behaviour of economic actors is purposeful. Actors have goals whose accomplishment they pursue. In that pursuit they build and follow rules and procedures, ‘policies’, which reflect adequate calculations but are not necessarily optimal in the neo-classical sense.
• One major goal of economic actors is ‘profit seeking’, they follow “policies” whose profitability they “inexactly compare, from time to time, with individual alternatives that present themselves by processes not entirely under their control”.^2^

Competition is an important factor that affects the decision-making process of economic actors. Businesses constantly make decisions affecting their future. Hamel and Prahalad (1994) argue that competition for the future, strategic competition, is about ‘opportunity share’ in contrast to competition for the present, which is concerned with market share and operational efficiency. New market niches are even more unclear in their operation compared to existing markets. Therefore, strategies to capture future opportunity share are not driven by the anticipation of immediate financial returns but by the prospect of having a leading position in future industry structure.

It is not surprising therefore that Hamel and Prahalad also argue that very often it is ‘contrarian’ companies that excel in this strategic competition. The contrarians are firms that will challenge the ‘orthodoxy’ i.e. the established way of doing things, the managerial and wider social ‘mental frames: “To discover the future it is not necessary to be a visionary, but it is absolutely vital to be unorthodox” (Hamel & Prahalad, 1994, p. 99). Companies have to dream of products not yet created and which by definition do not reflect customer surveys since it is extremely difficult for customers to imagine what is feasible. Not only has the firm to dream about the future (strategic intent), it also has to devise the way to get there. “Strategic architecture” is “…a blueprint for how to turn the dream into reality” (Hamel & Prahalad, 1994, p. 107). In order to do so, the firm has to ‘unlearn’ and replace the part of its past that does not usefully serve its ‘blueprint’.

Several factors have changed in tandem to the changes in government policy since the early 1990s (reflecting global cultural and ideological shifts) most importantly long term market growth prospects; product, process and marketing innovation and other government policies (like environmental legislation). When faced with this type of changes and the uncertainty they entail, many companies are reluctant to exit markets that still appear profitable or to abandon well-established practices and technologies that may however be unsuitable for the new business reality. Barlow and Duncan (1994) as well as Barlow and King (1992) argue that the high degree of uncertainty inherent in British speculative housebuilding forces companies to stick to norms, strategies and positions that are suboptimal. Ball's argument is that it would be very difficult for any housebuilding company to survive without the capacity to manage the uncertainty inherent at all levels and phases of development (see for example Ball, 1999). However, it could be argued that one way to tackle uncertain conditions would be to innovate so as to exploit the opportunities they offer. Therein arguably lies a key to competitive advantage and to long term firm survival and growth.

In any type of land development, as Byrne (1996) has noted, there are certain tasks involved: future demand has to be estimated in terms of quantity and quality/type, sites have to be identified and secured to satisfy this demand, the same sites have to be designed and planned to meet this demand, finance has to be found in order to fund acquisitions and construction, the whole process of design and construction has to be coordinated and managed and the end product has to be sold or let and managed/maintained after that. He thereafter separates development in four stages: Appraisal, Acquisition, Production/Construction and Disposal and identifies the uncertainties inherent in these processes. This list of functions is very close to de Magalhaes’ conceptualisation of agents and functions (de Magalhaes, 1996, 1998, 1999).

Byrne elaborates that the sources of uncertainty in the appraisal stage have to do with changes or lack of clarity in the project's specifications or other parameters, not realistic assumptions about aspects of the project as well as the market and not clearly specified objectives and design characteristics. In acquisition the biggest source of uncertainty is the response of the planning authority to the demands of the developer therefore usually the final purchase of the land is done after planning permission is granted. This means that profits are squeezed because a high price is paid for the land. During production, uncertainty arises around the project's characteristics therefore the more details are finalised prior to construction the less uncertain the project becomes, it is also easier to standardise its production hence costs drop. Finally, in disposal, the final product is released to the market and the original assumptions are tested. Rent/prices and investment yields are the biggest source of uncertainty in that process.

Detailed descriptions and analyses of housing provision and production in the UK can be found amongst others in the work of Ball (1983), Gruneberg and Ive (2000a, 2000b), Adams and Watkins (2002), and Barlow and Duncan (1994). Between them, they argue that the main structure is ‘speculative’, covering the private owner–occupier market, complemented by a residualised ‘contract’ structure providing for the social housing sector. It has also been argued elsewhere (Adams, 2004; Breheny, 1998; Karadimitriou, 2003, 2005) that there are strong indications of the shift of housebuilding in the UK towards PDL redevelopment from a predominantly expansionary process, geared towards building on new, undeveloped land.

According to Adams and Watkins (2002, p. 129), housebuilders have to become exceptionally good at deploying special skills in three generic functions: Landbanking/land management, planning and marketing in order to make greenfield speculative housebuilding feasible. These skills, institutionalised through norms and routines, also reflect areas where the firm integrates labour, land, money and knowledge inputs. Housebuilding can also be viewed as an activity with two distinct aspects to it; namely development/wholesale and construction (Wellings, 2006). It is through development/wholesale that traditionally the higher margins are achieved in the industry, it is also the part which carries the biggest uncertainties. Construction on the other hand usually is a lower margin activity than can be disassociated from housebuilding as such, housebuilders are not primarily construction companies and indeed as Wellings points out (2006) mergers between construction companies and housebuilders are rarely successful.

Based on the above rationale and assuming that there is actually a difference between non-PDL and PDL housebuilding, it could be argued that the ‘greenfield/expansionist’ and ‘brownfield/consolidation’ approaches to urban growth could give rise to several different alternatives to organising the housebuilding development process. This variety would be generated to a large extend by the great number of uncertainties involved in that process and the different ways businesses can respond to that uncertainty. The actors involved in each alternative will have developed their own norms, routines, skills and practices, their individual ‘genetic coding’.

Changes in government policy that relate to the types of land input used by housebuilders pose a significant challenge to the industry. In order to be able to adjust to this environment a company would have to strategically reorient itself. It is the adoption of norms, routines, skills and practices that are more suitable for the business environment which determines the survival of housebuilding firms but it would be impossible for a company to develop these without strategic commitment and intent, very often deriving from a contrarian approach. Having said that, if the adaptation is successful it may well act as an example of imitators. A successful adaptation will enhance the competitive advantage of those who adopt it, allowing them to survive and grow, thus changing their relative position within the industry. To the extent that this adaptation is linked to qualitative changes in output, its diffusion will lead to changes in the aggregate output of the industry as a whole.

## 3 The changing face of housebuilding

This section elaborates on the housebuilder responses regarding the way they organise and execute the production of housing developments and the type of spaces they are producing. It examines the changes occurring at the macro level, for the industry as a whole. Whereas this section elaborates on the industry's response at the aggregate level, Sections 4 and 5 will explore the strategic responses of the Berkeley Group and George Wimpey (now Taylor Wimpey, hereafter referred to as Wimpey) towards the new policy regime and its implementation.

The analysis in this section enhances, with the benefit of hindsight, the arguments first set out in Karadimitriou (2003, 2005) and Adams (2004). It was argued then that in response to the qualitative and quantitative change to the land input into the production process the industry has changed the types of dwellings and developments produced until the mid-1990s. Housebuilding in the UK was mainly based on expansion into the ‘greenfields’, therefore housebuilders have developed certain skills, norms and routines permeating the “structure of organisation of agency relationships” (de Magalhaes, 1996) that underlies housebuilding production processes. Landbanking and land management, planning and marketing have been the foci of recent literature as the three ‘generic’ skills required for housebuilders to be successful (Adams & Watkins, 2002). The configuration of spaces, the developments, produced by this structure of agency relationships are low density housing developments, marketed on the basis of their environmental amenity and aiming at customers seeking family-friendly built environments.

Several interviewees confirmed the conceptualisation of the 3 generic functions (Adams & Watkins, 2002) when they referred to the crucial elements of a housebuilding business. Many interviewees also confirmed that in housebuilding a great deal depends on the speed of transformation from site purchase, to building, to next site purchase (see also DCLG, 2007). One of them, a person involved in depth into the strategic planning and landbanking practices of housebuilding, said that the basic capacities of housebuilding firm are:

Ability in securing sites, in transforming acquisition into planning permission as quickly as possible, in marketing and selling houses and in buying a new site.

The first thing to note is that policy-induced changes in the amount and the type of the land released from the planning system for housing has increased the proportion of PDL used for housebuilding. This change is linked at least to two types of changes in the qualitative aspects of output until 2007: (i) the substantial rise in the proportion of flats as a percentage of new dwelling production and (ii) the significant increase on average in the densities of new developments (Karadimitriou, 2005). Since trend (i) is slowly reversing since 2008, dwelling production could be seen as comprising two generic product range categories, one based on low density more land-intensive development and another based on high density more capital-intensive development.

The change in output however also reflects changes in the nature of the generic skills required for housebuilders to stay in business. The traditional strategic planning and landbanking capabilities had to be complemented by flexibility in design and construction and the capacity to ‘negotiate’ throughout the development process. Marketing has also changed its focus as the vibrancy of urban living re-emerged as a key marketing theme during the 2000s. This enhanced flexibility and the new marketing focus are underpinning the long term trend towards specialisation that previous research has identified (Barlow, 2000). Furthermore the ability to provide higher levels of customisation through a more flexible approach also taps into significant latent demand for more customer input in dwelling production (Barlow, 2000).

Wellings (2002) as well as Nicol and Hooper (1999) indicate that from the 1990s onwards the trend of housebuilding industry consolidation has accelerated and several leading firms have strived to gain market share and to achieve output growth through Mergers and Acquisitions (M&As) which, as mentioned in Section 2, may well indicate increased ‘competition for the present’ in the absence of strong strategic visioning (Hamel & Prahalad, 1994). This trend continued throughout the 2000s and some of the most impressive deals happened just as the market was beginning to turn, for example Wilson Bowden's acquisition by Barratt for £2.2bn, the purchase of Crest Nicholson by a consortium of HBOS and Sir Tom Hunter for £715m and a similar deal between McCarthy & Stone and HBOS for £1.1bn.

**Fig. 3.1:**
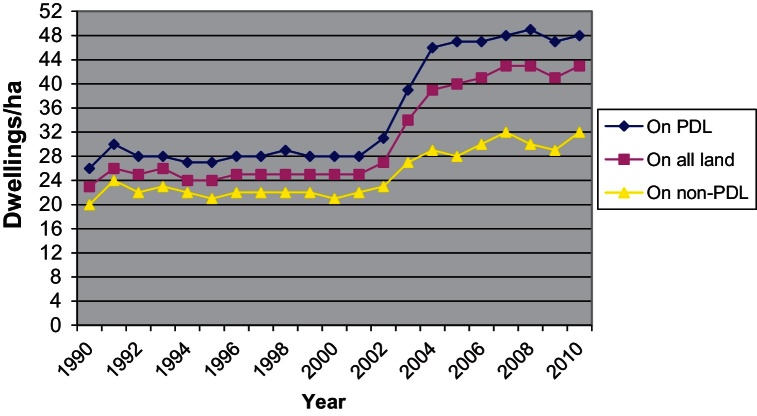
Average density of dwellings in new developments, England 1990–2010.

*Source*: Adapted from Land Use Change Statistics in England (DETR, 1998a; DTLR, 2001; ODPM, 2002, 2003b, 2005a, 2006; DCLG, 2010).

The upward turn in the percentage of dwellings built on PDL throughout most of the 2000s was combined with a marked increase in the average density of newly built developments which also begun to pick up considerably since 2002, almost a decade after densification made its first appearance as a policy direction, and has stabilised between 40 and 44 dwellings per hectare since 2007. This level is around 70% higher than that of 2001 (Fig. 3.1).

**Fig. 3.2:**
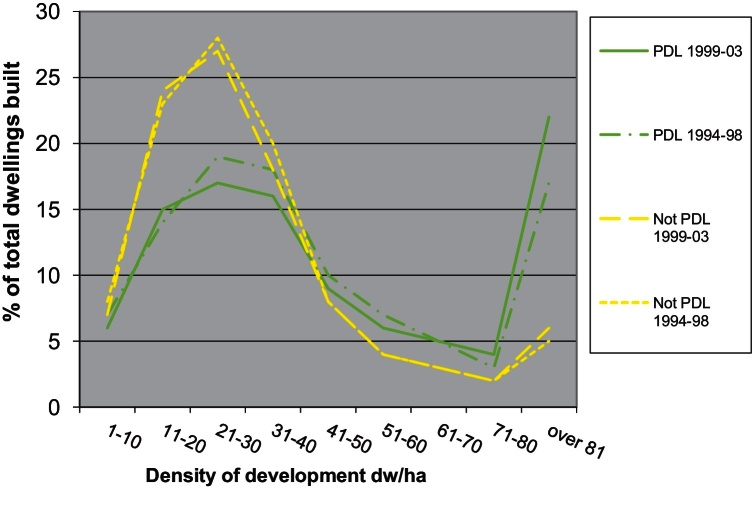
Average % of dwellings built by density category and type of land, England 1994–2003.

*Source*: Adapted from ODPM (2004).

More evidence pointing to the process of differentiation of the type of developments produced depending on the type of land used, comes from further analysis of the distribution of housing output per density category (Fig. 3.2) for the years 1994–2003. Unfortunately it was not possible to find data for later periods so this analysis can only present a specific trend which occurred during a time when the policy push towards densification was gaining momentum (see Karadimitriou, 2005). Of all dwellings built on non-PDL during the period 1999–2003, 58% where built in densities below 30 dw/ha and 76% where built at densities below 40 dw/ha. Only 6% of the dwellings built on non-PDL where built in schemes with densities above 81 dw/ha. More importantly, this distribution has not changed significantly between 1994 and 2003 which indicates a relatively stable development model (low density non-PDL configuration).

On the other hand, a significant 22% of total dwelling output on PDL was built in developments of 81 dw/ha or more during the period 1999–2003, up from 17% in 1994–1998. In contrast to that, 38% of dwellings built on PDL during the period 1999–2003 were below the 30 dw/ha mark rising to a cumulative 54% below the 40 dw/ha mark, down 4% compared to 1994–1998. This shows that whereas non-PDL is linked to low density development there was in late 90s and early 2000s a strengthening development model using PDL for higher density development (high density PDL configuration). The side effect of the trends shown in Fig. 3.2 was the increase in the polarisation of output mainly due to the increase in high density PDL output and decrease in low density PDL output. Table 1 describes in more detail how this polarisation evolved with time during the late 1990s and up to 2003. Whereas a significant percentage of total dwelling output was produced as part of low density developments, dwelling output in high density developments did also increase significantly. Output in medium density developments between 30 and 80 dw/ha was steady on non-PDL land and was dropping on PDL land.

Medium density PDL (30–80 dw/ha) is also an option but was not a rapidly growing trend probably because of its lower profitability compared to high density PDL. The share of medium density non-PDL during the period 1994–2003 was dropping as a percentage of dwellings built.

**Fig. 3.3:**
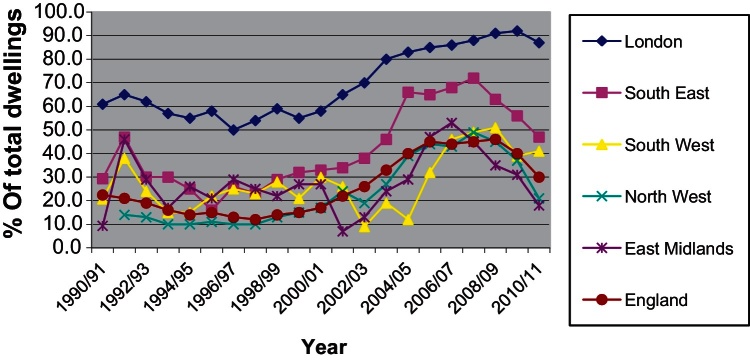
Flats as % of total private dwellings built in selected English regions, 1990–2010.

*Source*: DCLG, Live tables on house building, Table 254.

Looking at the composition of output one can observe that the percentage of newly built flats begun to rise in 1997 following several years of sustained decline (Fig. 3.3). Until 1997 the proportion of houses in the total amount of dwellings built was increasing from year to year. However, between 1997 and 2008, increasing densities and increasing percentage of flats built indicate a changing balance between types of development produced. These outputs can only be achieved by multi-storey apartment building although there is still ample latitude in the variation of feasible configurations of developments.

After 2008 the percentage of flats in total private dwelling output has dropped from 46% in 2008/2009 to 30% in 2010/2011 while during the same period the percentage of 3 and 4+ bedroom houses has risen from 46% to 60%. Given that the average density of development has remained stable since 2004 for both PDL and non-PDL (Fig. 3.1) it would be interesting to explore further what the situation currently is regarding the dwelling output per density category and type of land used (i.e. the distribution of output between development configurations as shown in Fig. 3.2).

Whatever the case may be it is useful to note here that Fig. 3.3 shows the change in the composition of output has only marginally affected London whereas it is very apparent in many English regions. Similarly, according to the same DCLG data, private dwelling production in London has dropped by 22% since 2007 compared to a drop of 46% for England as a whole. Thus in 2010/2011 London accounted for around 14% of private dwelling production (and 17% of total dwelling production) in England, up from 10% (and 13% respectively) in 2007/2008. The fact that one in six dwellings in England in 2010/2011 was built in London and around 90% of that production was flats should have an effect in aggregate density and housebuilding statistics for England.

As a result of the changes in government policy related to the supply of land, housebuilding firms were faced with a requirement to significantly change their strategies and to develop new know-how in order to respond to the changing circumstances of their business environment. Not all firms interpreted that change in the same way, nor have they responded to it similarly. However, housebuilders are faced with limited strategic options when faced with the constraints imposed by the planning system in terms of land inputs. Though the latest trends in dwelling production indicate a geographical dimension to firm differentiation (London diverging from the rest of the country), the changing land input had significant effects on the various “structures of organisation of agency relationships” underlying private housebuilding and on the organisation of production (de Magalhaes, 1996).

As the use of PDL was increasing so was the percentage of new dwellings built on PDL and the percentage of these new dwellings that were flats. This however also means that the built environment produced as part of that shift had significant differences in its configuration from the environments previously produced. In order to produce these new configurations of spaces the actors involved had to reconfigure the way they organised their production processes. As the analysis above shows, one of the most significant changes that occurred in housebuilding is the densification of non-PDL developments and the boost of production in high density PDL developments of above 80 dw/ha. Following the market downturn, flat production has disproportionately declined outside London, raising interesting questions as to why high density schemes would be deemed unviable whereas low density schemes would continue to be built.

These questions however could be the inspiration for further research in the future. The section that follows will examine in detail the generic strategies and practices of two top London housebuilders first discussed in the literature in mid-2000 (Adams, 2004; Karadimitriou, 2005), the Berkeley Group and Wimpey (George Wimpey prior to 2007 and Taylor Wimpey thereafter).

## 4 Housebuilder decision making

The previous section argued that dwelling production in the UK evolved towards polarisation insofar as the types of developments produced are concerned. It showed that following the end of the housing boom, some housebuilders are shifting their production towards multi bedroom houses whereas one-bedroom flat production has declined. In the previous section, the hypothesis was also made that the types of built environment produced still cater for at least two different types of demand, one for low density (mainly on non-PDL) and the other for high density apartment living (mainly on PDL). Apart from an apparent geographical differentiation between London and the rest of England, this polarisation may well reflect wider inequalities in income distribution and associated spending power. However in order to understand the situation from the point of view of the housebuilding firm it is worth looking into housebuilders’ strategic options when it comes to the potential land type/density mix they are faced with.

So far as the type of land used and the density of development produced are concerned, housebuilders have essentially six alternative ideal-type combinations as strategic options to choose from, shown in Table 2.

The professionals involved in the non-PDL low density housebuilding process, from architects and planners to quantity surveyors and marketing experts have become accustomed to working with this type of land and the products that go with it. Their expertise has developed in particular ways, institutions, norms and routines have evolved in order to standardise the process and increase efficiency. This specialisation has developed to such an extent that when asked if any aspects of non-PDL housebuilding and building on PDL are similar a senior manager of a major housebuilder said: “No, they’re not, it's a different industry”.

When asked how he would call the types of development his company are building the same interviewee stressed the differences in the element of risk involved in PDL high density housebuilding compared to non-PDL low density housebuilding:

It is regeneration. We are building private homes and affordable homes in fairly close proximity and that has risk attached to it…To actually build a new community and sell expensive flats to people to create a cross subsidy to build cheap flats and bring poor people next door, is a risk that the rich people might say ‘not for me thank you’…It is a risk having to build commercial shell space and wait perhaps years until anybody wants it because they are waiting for a critical mass of population to use their shops, their restaurants, their gymnasium or whatever.

Additionally, the change to high density PDL development posed new challenges for the industry with regard to the response of the planning system. In the words of a senior manager familiar with the planning aspects of housebuilding:

…the industry as a whole, suffer from the same setback. They may achieve all of the objectives (design, parking, density, etc.) but local authorities do not follow government policy and stop the implementation of the new standards. This is reflected in the high number of appeals, council members decide opposite of government advice.

This was at a time when government was pushing for denser development. More recently, the introduction of the Localism Bill (now an Act) seems to have diversified these views. Land Securities for example anticipates that localism will exacerbate anti-development sentiments in urban areas like London but may actually reduce resistance to development in more rural areas (Land Securities, 2011). According to senior managers from Berkeley and Wimpey interviewed for this research, the cost of purchasing non-PDL land is a major element of the total cost of development, reaching 40% in some cases thus making land speculation extremely important for overall profitability. This should be compared to around 10% in high density PDL developments, as evidenced for example from Berkeley's own estimates. In 2009 Berkeley's average dwelling sales price was £395,000 whereas their average plot acquisition cost was £33,000 i.e. 8.3% of the average dwelling sales price. As one interviewee eloquently said when asked how profits are made in high density PDL housebuilding:

We know how to work the density. Land value is a fairly low percentage of development value and if you are buying a green field you might pay 35% or even 40% of the value,… [in a PDL]… it is between 5–10%, so yes, the land might be £30 million and that is a lot of money to pay the interest on every week. It goes through a cycle where the borrowings might go up to £100 million but when it comes down, if you are a long term player running a public company and if at the same time you have another project that started back here and has peaked and come down here, so if you look at the bigger picture it evens out so land values are not that significant.

This difference in percentages also reflects the relatively moderate cost per square metre of non-PDL housing construction and land servicing. However, it highlights the importance of landbanking and land management as a cost control mechanism and hints at the potentially higher margins that housebuilders with good landbanking skills could enjoy for upmarket non-PDL developments, especially highly priced detached houses. Indeed, when 30–40% of costs are land costs then margins will be more affected by changes in this cost element.

Thus, for Wimpey and for many other housebuilders gaining planning permission for a site should be better seen as a process of transferring the development rights from the state/social sphere to the private sphere. It is a process which, as Ball argued in 1983 and as is evident from the paragraphs above, in the case of non-PDL housebuilding starts with the efforts of housebuilders to influence the planning system to designate land in accordance with their strategic landbank holdings during the development plan formulation process. This, in turn, requires substantial land market know how and planning capabilities which are usually area-specific thus forcing housebuilders to maintain some degree of geographical fragmentation both as individual companies and as a sector, a trait well explored by Ball (1983). This set of skills is quite different from the negotiation skills required to see a planning application through the planning system and to constantly revise it thereafter.

Government policy from the early 1990s onward had the consequence of reducing the importance of out of town non-PDL strategic land and strategic planning knowledge, at least in geographical terms and for as long as PDL land was the order of the day. By the same token it made negotiation, consultation, partnering and land consolidation skills more important. The cards have been reshuffled once more following the turn towards localism and the relaxation of government policy towards expansion witnessed in the NPPF. Planning negotiation skills continue to be important but greenfield strategic landbanking capacities also gain in significance, thus major housebuilders who wish to have broad geographical coverage may well respond with an increase in the variation of their approaches to development in order to reflect the variety of their business environment.

The point is that changes in land input require a change in approach from developers and the planning system alike and are eventually reflected on housebuilder landbanks and the type of dwelling output. Partially because of the powerful sustainability discourse and partially because of practical, locational, reasons, many PDL sites were allocated as potential development sites from the Local Planning Authorities (LPAs). PPG3 and the sequential approach had put extra pressure on both the developers and the planning system to promote PDL site redevelopment, whereas PPG4 and PPG13 also tried to influence commercial development and transport infrastructure in the same way. The introduction of the Strategic Housing Land Availability Assessment (SHLAA) following PPS 3 of 2006 and the obligation of Local Authorities to satisfy stringent housing production and land utilisation goals further elaborated the tools aimed at regulating housing production and land used.

Similarly, the requirements for Statements of Community Involvement following the Planning Act of 2004 added an important dimension to the process by highlighting the role of community consultation prior to any property development project. This element will be further enhanced with the shift towards Neighbourhood Planning which is now taking form whereas the replacement of the PPS-RSS system with the NPPF and the ‘duty to cooperate’ introduces an element of greater flexibility without necessarily changing the overall strategic goal of reinforcing sustainable urban development (thus still promoting densification up to an extent). What is actually going to happen insofar as dwelling output is concerned is anyone's guess at the moment. This ‘wait and see’ approach is reflected in the latest annual reports of both Berkeley and Wimpey.

With regard to planning skills, there still seems to be scope for developers of PDL to put pressure on the planning system during the plan-making process in order to allocate developable PDL land according to their landbank. This ‘tweaking’ of the system however is a crucial element in non-PDL development too because in many cases the LPAs are rather negatively predisposed in releasing ‘greenfield’ land, partially reflecting pressures from local constituencies. Therefore, the assertion by Land Securities that development may become easier outside the big urban centres may sound surprising at first. However, it has to be seen in conjunction with the New Homes Bonus, which in principle at least should provide a strong incentive for local authorities to allow more housebuilding within their boundaries. The shift towards ever increasing community participation in turn makes stakeholder engagement skills and planning gain negotiation skills on behalf of the developer much more important. These ‘collaborative’ skills, which accommodate for the constant change of the development's design and mix of uses are also essential for other actors, like the LPA and the local community.

An interviewee involved in the planning process for both PDL and non-PDL schemes said that although all developers are “reasonably constructive”, Berkeley followed an impressive cooperative attitude quite different from the industry standard. Berkeley themselves are quite aware of this and very much in favour of the latest Localism Act requirement for pre-application consultation. Similarly, Wimpey's approach seems to have changed in the years after the merger with Taylor Woodrow and is now also recognising the importance of consultation with LPAs and local communities. This of course does not mean that these housebuilders will not make use of the legal means available to them, i.e. ‘double track’ or appeal, as demonstrated by the case studies. With that in mind, the interviews conducted as part of the case studies indicate that the planning system is often treated by housebuilders as a process that needs speeding up in order to speed up the production cycle. This ‘need for speed’ is reflected in much of the discourse underpinning the linkup of LPA funding with planning permission determination time and with the constant efforts to reform the planning system with a view to ‘speeding it up’.

PDL developers face different uncertainties when compared to non-PDL developers. Granting development rights on PDL should in principle face less planning-induced resistance given that policy is in principle still promoting consolidated urban growth and there are few if any established local communities in and around most PDL sites. For example Wandsworth, where the case studies are situated, prided itself throughout the 2000s for being an authority with a can-do mentality which welcomes development and whose approach according to a person knowledgeable of its planning approach is “to maximise the benefits of the use of land”.

Other than that, critical views of PDL redevelopment emphasise the uncertainty over development coming from the material conditions on the site itself (contamination, existing structures requiring demolition, etc.). However, as the interviews have revealed, these issues are of a technical nature and a manageable risk for companies which have developed suitable know-how. This view has been confirmed by surveys (Shephard & Dixon, 2004) showing that technical issues like contamination are not deterring adequately resourced developers.

Finally, in terms of marketing the development, the importance of attracting customers by selling a lifestyle more than a product should not be underestimated. It should not come as a surprise then that a new marketing approach has been deployed to promote PDL high density developments. The marketing of low density developments and dwellings usually built on non-PDL very much rotates around the ‘idyllic countryside living’/’escape the city evils’ theme. The way the urban renaissance/sustainable community discourse was expressed in the greenfields vs. brownfields dichotomy increased the difficulty of successfully marketing developments on PDL based on the ‘rural idyll’ marketing model.

The lifestyle sold with an urban PDL high density development therefore contrasts sharply to the lifestyle sold with a low density development, either PDL or more often non-PDL. In urban PDL developments priority is given in promoting the image of the ‘trendy’, ‘urban chic’ and ‘vibrant’ character of city living. Images of young, dynamic (often single) and trendy individuals are evoked to promote the ‘aspirational status’ of the clientele. The architectural and design features of the buildings are contemporary as opposed to the traditionalist features of low density non-PDL ‘rural idyll’ housing. This target group usually has enough disposable income to pay for the prices that this strategy of product differentiation entails.

From the discussion and the analysis thus far it emerges that during the last dozen or so years, housing provision in the UK went through an ‘adaptation’ phase. Non-PDL land used for housebuilding in 2009 was 31% of the total land used for housebuilding (down from 54% in 1994) and in 2010 the percentage of new dwellings built on PDL stood at 76%, down from 80% in 2009 but up from 54% in 1994. This change in the type of land used had an important effect on the types of dwellings produced. The higher the percentage of newly built dwellings on PDL the higher the percentage of these new dwellings that are flats. More flats meant more apartment blocks which in turn implied denser development formats. At the same time, total non-PDL land consumption was dropping. Until 2007 this could be attributed to the turn away from land-intensive non-PDL development, thereafter it is also associated with the overall collapse of housing production and the densification of non-PDL development. Production output has not recovered since and in all likelihood it will not recover substantially until the current property market conundrum is resolved.

This trend towards densification had an effect on the generic skills required for success in housebuilding so far as PDL is concerned. The locational and qualitative requirements of PDL housebuilding require the restructuring of existing landbanks and incur an opportunity cost and actual resource commitment on behalf of housebuilders. Shifting the composition of landbanks once more towards more non-PDL sites in response to the requirements of localism would have a similar effect although it may well be the case that several housebuilders never really restructured their strategic non-PDL landbanks. At the same time, effective partnership-building and negotiation skills continue to grow in importance due to the long term involvement that non-PDL development requires from housebuilders. Long term involvement within an uncertain post credit-crunch environment also increases the importance of s.106 agreements or any other value extraction arrangement (CIL, etc.). Marketing is also dramatically affected. Selling lower density non-PDL developments is based on the ‘rural idyll’ ideal whereas high density PDL developments, or at least their urban variant, are sold on the merits of the ‘urban lifestyle’.

The following section will examine how Berkeley and Wimpey had to adapt to the changes in policy regime that started in the mid 1990s and the way the two companies weathered the crisis of the late 2000s. It will do so by looking at their strategic response to policy change, their norms, routines and skills at the operational level and at the performance of each company throughout the period in question.

## 5 Case studies

The previous section illuminated some of the changes shaping the industry at the aggregate output level and argued that there is a link between the changes in planning policy and the significant changes occurring in the types of dwellings produced and in the built environment thus created. This section will focus on the firm and on the development site level and will explore how the transformation of the end product is linked to a process of strategic adaptation by two major housebuilders to the new business environment.

Depending on the timescale and the type of response of each firm, this reorientation affected their competitive position which in turn affected their present and future profitability and their chances of survival and growth. Residential development is a business activity involving high degrees of uncertainty. There are uncertainties about the future market, consumer preferences, economic and fiscal policy, product construction and specification to name just a few. Firms can be short-sighted in the sense that they cannot fully anticipate the consequences of their actions let alone the shape and form of their future business environment. This section will look at some of the ways in which the two housebuilders organised themselves in view of this uncertain business environment.

### 5.1 Berkeley and Chelsea Bridge Wharf

#### 5.1.1 The Berkeley approach

In the case of the Berkeley Group, market uncertainty is managed through a flexible approach towards the planning and design of the product (i.e. mix variation, change in specification) made possible through a highly developed construction management approach that relies on inputs from a market feedback mechanism. This market feedback mechanism combines information about demand and supply conditions in different markets (retail, housing, office, leisure, etc.) but also what the characteristics of that demand are i.e. which types of products are more sought after. In that respect information from other divisions (i.e. Berkeley Commercial) is crucial as well as information from forward selling.

According to Tony Pidgley, MD of the Berkeley Group, the Group is in the business of “…adding value to land, through the application of our development skills” (Berkeley Group, 1992, p. 4). The process of land development and thus of adding value to land is split by Pidgley into five ‘functions’:

• land acquisition and use optimisation,
• planning permission,
• design of product,
• construction,
• marketing and sales.

These functions are treated as a constantly evolving process and therefore they are only separated here for analytical reasons. Site selection and acquisition are market-driven in the sense that local land acquisition teams try to verify whether a site has the potential to suit the needs of customers. Depending on the area that potential may derive from good schools, amenities, shopping opportunities, etc. Therefore, decentralised land acquisition by teams with excellent local knowledge is essential.

The following quote from Berkeley's 2010 annual report (p. 4) summarises their approach and has been reaffirmed in several interviews:

Maintaining our operating margin above 17% is a result of Berkeley's strategy where land buying is highly selective – driven by opportunity not volume – and where Berkeley has the time and expertise to add value to its land holdings.

A manager with deep knowledge of the practices of the Berkeley Group suggested that indeed what matters for the group is not how long a site stays in its possession but how much value is added by the company during the time that the site stays in its possession. In general, he added, Berkeley is “not intimidated” by holding sites for a long period and takes the time “to get most out of the site”. This statement reflects the attitude and the level of specialisation required by the very nature of the business that Berkeley is involved in: in 2004 the company withdrew from all non-PDL housebuilding and thereafter focused solely on ‘large scale complex regeneration’. This was combined with a share buyback scheme which in effect would increase the control of the management team over company affairs whilst returning significant amounts of capital (£12 per share) to shareholders within seven years (up to 2011). In 2008 and in view of deteriorating market conditions the company's AGM decided to defer the last payment of £3 to 2014 and instead use the funds for land purchases. One year later, the company focused even more on land purchases to be funded through an increase in share capital, the goal to return £3 by 2014 was abandoned in consultation with the shareholders.

The teams involved in land acquisition are also skilled in development. Their skill base ranges from surveying, to planning, to design, to marketing and they work together on a site from beginning to end even when external expertise is brought in. These ‘multiskilled’ teams not only manage the uncertainty of the construction process but also facilitate a more flexible approach to the design and the specification of the final product. Customer input can be used from the very beginning in combination with ‘forward selling’ practices. When Lai, Wang, and Zhou (2004) looked into techniques that can be employed to manage the uncertainties arising from the unpredictability of future demand they argued that:

…the presale method not only helps developers deal with the uncertainty of future demand…but can also substantially reduce developer's inventory costs… This method is particularly useful for large development projects…

In Berkeley developments, the layout of the flats or houses can be revised even at a late stage during the development process, to accommodate for changing market conditions and customer requirements. According to several interviewees, profit making through land trading was not within the strategic priorities of the company but emphasis was put on adding value to land through development. In post-2007 company annual reports, however, increased emphasis is placed on landbanking and indeed, as mentioned previously, the whole corporate strategy shifted in order to channel company resourses into land acquisitions.

Typically, in order to minimise market risk and improve cash flow and returns on their invested capital, housebuilders would aim at minimising the time that lapses between taking the decision to develop and selling the final product, their frequently aired dissatisfaction with the ‘slow’ planning application process testifies to how crucial the time parameter is. However, volume production of large, apartment block developments on PDL which are more complex in terms of design, planning and construction poses a significant challenge in terms of turnover time. Additionally, by virtue of their size, these developments can form a substantial part of a housebuilder's annual output thus increasing the exposure of the balance sheet to market risks. Flats in apartment blocks are difficult to occupy while the block is under construction. Thus, high density apartment block developments can potentially make housebuilders more vulnerable to changes in market conditions compared to traditional low density housing developments. Flexibility in the practices employed towards the timing of sales is key in tackling the risks that come with this new business reality. Therefore one way to release part of the block for sale earlier in the process is to engage in forward selling.

Forward selling is often used as a way to decrease the cash flow imbalances that typify speculative housebuilding. However, forward selling not only allows for a more balanced cash flow but also acts as an insight into market demand. This information, combined with sales information from the rest of the Group can be then translated into quantitative and qualitative alterations in supply and thus make it more responsive to fluctuations in demand. Finally, another big advantage of forward selling is that it allows the company to start work on the site “as soon as possible” but at the same time to be responsive to the demands of the clients by incorporating their preferences into the product. The developer is thus living up to the marketing strategy by selling a personalised product for which it can attract higher premiums. Although today forward sales are a practice widespread throughout the industry, the integration of the practice into the production process as well as the extent that this practice is used at Berkeley are exceptional. Indicatively, in 2003 ‘cash due on forward sales’ was £920.9 million compared with total sales per year of £1130.1 million (Perrins, 2003) whereas in 2007 forward sales amounted to £936.3 million compared to total revenue of £918.4 million (Berkeley Group, 2007) and in 2010 the cash due on forward sales was £648.1 million with total revenue for the year standing at £615.3 million (Berkeley Group, 2010).

Apart from forward selling, phasing is another important practice which aims at managing risk and profitably exploiting market fluctuations. When they looked into the benefits of mixed use schemes Childs, Riddiough, and Triantis (1996) concluded that they are useful in mitigating risks when tapping into uncorrelated markets and in adding economic value by pushing marginal revenues up when market oversupply in one type of use would otherwise have forced them down for any specific development. Depending on the efficiency of the construction process and the prevailing market conditions the size of each phase can vary from a few dozen units (one block of flats) to a couple of hundred units (several blocks of flats forming a neighbourhood). Thus production can be regulated and market risk can be controlled without compromising the long term completion of the whole development. The type, size and style of the apartments produced and eventually the market segment that each batch will be sold to are also subject to change. With the use of suitable construction methods (shell and core, fasttrack) the size and configuration of each batch of flats can be adjusted to suit market demand and the style of the apartments can be modified to fit the buyer's tastes and requirements.

One way to operationalise the mix of uses as a market risk management tool is to obtain a planning permission for a specific mix of uses but change this mix during the project's lifetime by submitting new applications. A variant of this approach is to obtain an outline planning permission which allows certain uses to expand or contract in terms of square metres but only within a pre-set band (so they cannot drop below a minimum and cannot rise above a maximum size). Therefore, under this approach, what was envisaged as a hotel in the original planning application may become an office block or an apartment block at a later stage and the total surface of each use may also change. Site planning and design become a unique process: sites are constantly re-planned with the aim of increasing densities “*…*whenever appropriate in line with best planning practice” (Berkeley Group, 2003, p. 9). This allows the company to increase output and profitability without additional land costs. This is by no means a practice unique to the Berkeley Group. A similar approach was taken for example in King's Cross where the outline planning permission defined the mix of uses in terms of a possible range of coverage that each use could occupy in the future. The way that arrangement would play out in practice was actually one of the main points of public concern during the Planning Committee meetings where the King's Cross Central development was discussed.

Change in the mix of uses combined with forward selling, flexible construction methods and appropriate phasing is a risk management approach comparable to the ‘stop-go’ approach used by traditional housebuilders and has the advantage that it potentially smoothens the cyclical variations in output. In the case of Berkeley it is complemented by collaborative working in multiskilled teams and often by a cooperative approach towards other stakeholders. When put together this set of practices could be called ‘the development management’ approach.

In summary, the main features of this approach include:

• Engaging in discussions with the planning authority early on, when the ideas about the project are still developing.
• Using multiskilled teams to tackle each site and keeping the same team involved throughout the process.
• Allowing planning, design and construction to overlap or run simultaneously instead of treating them in sequence to each other.
• Separating the development in autonomous phases; each phase could be built by a different contractor, under the supervision of the developer and its construction management consultant who coordinates the process.
• Varying the product mix for each phase based on the feedback from other corporate divisions and sales as well as forward selling.
• Approaching urban planning as an open-ended process linked to a constantly evolving scheme.

Elements of the ‘fasttrack’ construction method appropriately adapted to the UK context and to the particular circumstances of PDL redevelopment can also be used. Therefore, the overlap between planning, design and construction is combined with customer input and sales figures to allow appropriate modifications to each project phase even during its construction. This method is radically different to alternatives like the ‘Design & Build’ procurement but requires a much greater coordination effort. The outcome, however, is a build up of organisational capacity to manage effectively the uncertainties surrounding big, long term and complex projects.

#### 5.1.2 Berkeley's adaptation to policy changes

It is worth pointing out here that the strategic transition towards more PDL development was a gradual process of learning and adaptation for Berkeley. Given the government's policy focus the redevelopment of brownfield sites was seen as a move that would allow the group to exploit opportunities for future growth. The company gradually moved from its original identity as a builder focused on upmarket suburban housing (late 1980s–early 1990s) to large scale upmarket mixed use urban PDL developments, basing its business strategy on product differentiation.

The Group's transformation was neither sudden nor immediate but required long term commitment. The 1998–2001 period was a period of reorganisation for the group, as the 1999 annual report (Berkeley Group, 2000, p. 3) recognises:

The year has been a challenging one in terms of production. A growing number of the Group's projects are on brownfield urban sites which are more technically demanding thus requiring the right management teams in place with the right procedures and controls to deliver the finished units to the right timescale and costs. During the year Tony K. Pidgley has undertaken a review of the structure necessary to successfully manage the wide range and volume of developments undertaken by the Berkeley Homes companies. Accordingly, those companies have now been reorganised to ensure that our management teams, skills and expertise align more closely to the types of developments undertaken.

It seems that 1999 was a critical year in that process, which started in the early 1990s when Berkeley participated in the development of Brindley Place. That year's company report (Berkeley Group, 1999, p. 3) recognises that and also identifies a crucial element of the Group's approach towards redevelopment, the strict control over the development process.

A growing number of the Group's projects are on brownfield urban sites which are more technically demanding thus requiring the right management teams in place with the right procedures and controls to deliver the finished units to the right timescale and costs.

During that year the Group was restructured to “*…*align more closely to the types of developments undertaken” (Berkeley Group, 1999, p. 3). It seems that around that time the company realised that its future lay in urban PDL redevelopment, mostly in the inner city rather than the leafy suburbs. The following quote from the 1999 annual report is a good example of how Berkeley positioned itself in a broader business environment which the change in government policy was reshaping.

Our strategy was based on the fact that our development skills and expertise lent themselves most readily to expanding into city and town centres where we could concentrate on urban and brownfield schemes. This has proved very successful. Land supply is a key element of our business and obtaining the right planning permission within the right timescales is essential.

The Government has made it clear that it wants to see 60% of the new housing supply built on recycled or brownfield sites. These are precisely the sites on which the Berkeley Group is now concentrating and where it has acquired particular expertise. At the same time, the implosion back into city and town centres where people now enjoy living and want to buy their homes has generated an active and ready market for our products. Over the last few years, the Group has become skilled at developing complicated inner-city and town centre sites. Although these sites are not without their complications and frustrations, we believe that this expertise and understanding of the issues will contribute markedly to our future growth (Berkeley Group, 1999, p. 5).

However, several years after beginning its shift, the Berkeley Group still retained some production of executive suburban development as a residual activity. Its 2003 annual report vowed to “…remain committed to undertaking today's most exciting and challenging urban regeneration and renaissance projects” (Berkeley Group, 2003) and the same strong commitment appears to all annual reports up to 2010 whereby the policy uncertainty coming with the change in government seems to have led the company to tone down its references to regeneration whilst retaining the emphasis on sustainable community building.

The strategic review of 2003 led to the virtual abandonment of non-PDL housebuilding and regional activities thus leading to the divestment of Crosby Homes, the closure of Thirlstone and the withdrawal of Berkeley Homes from greenfield development. This major strategic re-orientation continued into 2004 with a proposal from the Board to phase out non-PDL housebuilding, scale down the business and gradually buy out existing institutional investor stakes to turn a group with a market capitalisation of £1.4 billion into a specialised housebuilder with a market capitalisation of about £500 million. After the restructuring, which was cut short following the 2007 downturn, Tony Pidgley together with three more directors owned a bigger percentage of the new company (the aim was for 15%). The following excerpt from the press release announcing the restructuring plan illustrates the unique characteristics of Berkeley and the important elements of the business model of the group. It also sums up the rationale underlying the processes of strategic adaptation that Berkeley has followed for the last two and a half decades, a rationale which is consistently transformed into action to the extent that this research could explore.

Berkeley operates a different business model to the majority of other house-builders as it concentrates mainly on highly complex, large-scale, inner-city, urban regeneration schemes on brownfield land where it can create enhanced returns for Shareholders and deliver benefits for all stakeholders. The strategic review… sought to assess the best route for delivering shareholder value. This took place in the context of the Board's views about the outlook for achieving sustainable growth in the markets in which it operates – where there appears to be a natural size for a residential urban regenerator – and takes into account the normalisation of the housing market following a decade of boom and a number of other external factors.

The Board considered a number of strategic options including continuing to grow the business, which required further investment and additional management teams, disposing of the business or selling-off or demerging individual divisions…After careful consideration the Board has decided to leave behind Berkeley's traditional housebuilding heritage and focus primarily on larger scale complex regeneration. This strategy allows the return of substantial capital to Shareholders while enabling Berkeley to continue to buy land selectively when attractive opportunities arise in the urban regeneration market. Critically, it is a path that will retain staff to ensure the sustainability and future of the business with the main challenge now being to realise the value contained within Berkeley's strong land bank.

This reorganisation not only sacrificed non-PDL housebuilding and wider scope and geographical coverage in favour of a more specialised company but also set the company on course for a management buyback of its shares. Reduced influence from the City had the advantage that it allowed the company to focus on the long term and on activities that the management believed it did best. That review also led to a renewed emphasis on other activities (affordable housing, commercial property, strategic land) that blended better with the business model and were highlighted as future growth areas.

As it turns out, the capacities to engage in affordable housing and in strategic land purchases where important in reducing the effects of the 2007 property market downturn on output, cashflow and profitability. However, Berkeley as most other housebuilders adopted a ‘wait and see’ approach following the change in government in 2010. Since then its annual reports emphasise concepts like sustainability and community consultation and tone down the emphasis on regeneration and PDL development.

#### 5.1.3 Berkeley's performance

The outcome of this long term strategic approach to development is that the company first grew at a dramatic pace and then managed to survive and stay profitable even during the downturn. Its output and turnover were increasing steadily prior to the downturn, its profit margins (ranging from 17% to over 20%) were steadily on or above the industry average. It did not engage in Mergers and Acquisitions (M&As) in order to maintain or increase market share or gain skills or assets. The evolution of the group is ‘organic’, based on sound financial practices, efficient operations and strategic vision that allows it to tap early on into emerging markets. Today, the company specialises in upmarket, high margin, mixed use developments in prominent locations and thus is active in relatively few markets (geographically).

The industry ‘league tables’ from EmapGlenigan/Housebuilder magazine show that already in 2003, the Berkeley Group was the top housebuilder company in London, based on applications to build, followed by a distant second Wimpey (Menary, 2003). In comparison, for the UK where the competition could build on non-PDL, Berkeley came behind Wimpey, Barratt, Persimmon and Taylor Woodrow, despite its limited geographic coverage (Wellings, 2002). The Glenigan/Housebuilder magazine league table for 2011 shows that in effect Berkeley has become an oligopoly in London but has withdrawn from all other markets except from the South East. Out of a total of 6912 units for London as a whole, Berkeley had submitted detailed plans for developments totalling 3989 units followed by a long second Asprey, with 480 units, and Barratt in third spot with 223 units (Menary, 2011). In the rest of the UK it is Persimmon, Taylor Wimpey, Barratt and Bellway who are leading the tables.

**Fig. 5.1:**
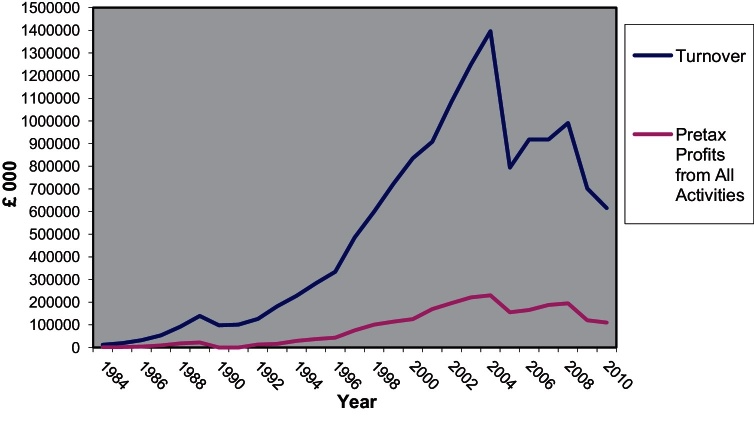
Berkeley Group, annual turnover and profits 1984–2010.

*Source*: Selected company annual reports 1984–2010.

Since the 1989–1991 market downturn, which caused a temporary slump in profits and turnover and up to 2004, Berkeley has gone from strength to strength. It has consistently posted double digit rates of annual turnover and profit growth^3^ with one exception in 1991 which can be attributed to the adverse market conditions at the time. It is characteristic that in 1992 its profits from housebuilding soared by 5153.7% compared to 1991 (Fig. 5.1).

**Fig. 5.2:**
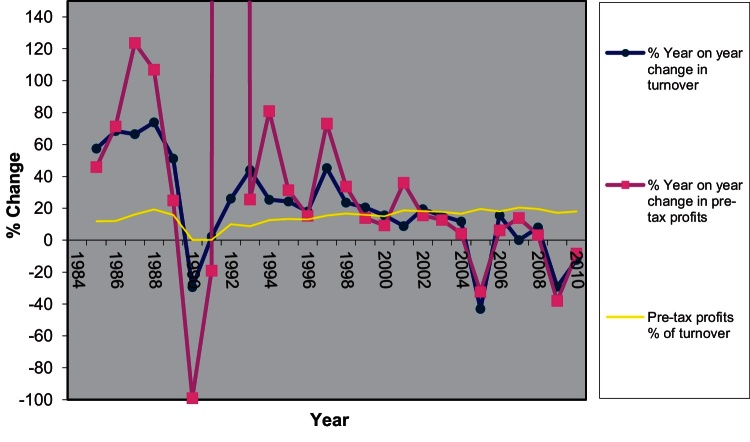
Berkeley Group, nominal changes in turnover, profit and profit margin, 1984–2010.

*Source*: Selected company annual reports 1984–2010.

However, even in 1989–1991 and 2007–2010 the company remained profitable while its competitors were facing losses (see the following section on Wimpey) (Fig. 5.2).

Apart from years when crises strike the only other years where profits from housebuilding declined compared to the years before were 2000 and 2004–2005. Interestingly enough, profit margins have been on the rise throughout the last 20 years and have remained above industry average even during the latest market downturn. In 2000 a slightly higher percentage of the group's profits came from commercial developments, in mixed-use schemes. In 2004 it was the Joint Venture activity (which included some Social Housing schemes) that acted as a buffer, although the group's performance in 2005 was affected to a large extent by the decision to totally withdraw from non-PDL housebuilding by selling Crosby Homes to Lend Lease and at the same time to adopt new accounting standards.

**Fig. 5.3:**
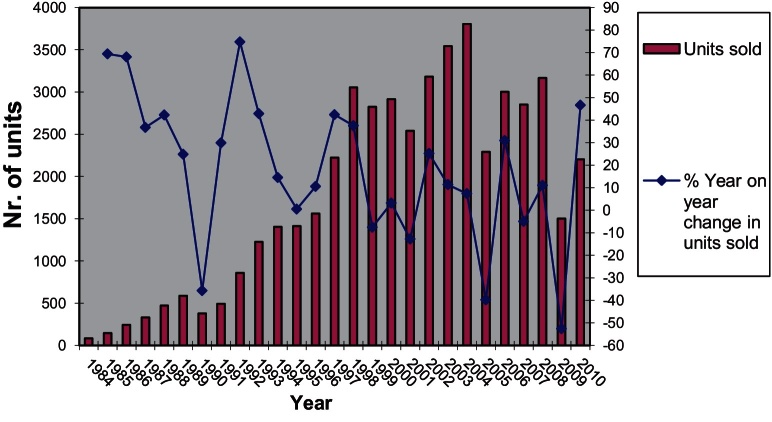
Berkeley Group, nr of units sold and year on year change, 1984–2010.

*Source*: Selected company annual reports 1984–2010.

In a mature business sector, like housebuilding, persistently high margins are a strong indication that the company's growth is not just an outcome of the buoyant property market but has to do with inherently efficient operations and good corporate capacity to tap into in high-margin markets via processes of strategic adaptation. A review of the corporate accounts, an example is shown in the quotes above, reveals that operational efficiency is a consideration but not what drives business strategy. It has to be noted here that this exceptional financial performance is accompanied by an equally impressive housing sales record, which however has some ‘bad’ years, coinciding with adverse market conditions and/or periods of corporate restructuring (Fig. 5.3).

This performance points that the company, through its strategic orientation and its organisation of the production process has differentiated its product enough to create what in effect is its own market and is exploiting its dominant position accordingly. How this was done will be further illuminated in the pages that immediately follow.

#### 5.1.4 Berkeley in action: Chelsea Bridge Wharf

Bearing in mind Barlow's comment that housing “…remains an essentially mass produced product, manufactured by using craft skills” (Barlow, 1999, p. 25) one can see in the preceding sections how the shift towards high density PDL developments created the preconditions for radically changing the way the built environment was produced. Although because of the nature of its methodology this research cannot comment on industry-wide trends in terms of production methods, it has uncovered substantial evidence that Berkeley changed the types of dwellings they were producing as well as the way they were producing them. This case study will explain this ‘way of doing things’ in more detail and will demonstrate how it was applied during the development of a specific scheme. The case study project, Chelsea Bridge Wharf, is a typical example of a Berkeley Homes approach. It is a large (approximately 900-unit), multi-phased, high density, mixed use development scheme which took a more than a decade to complete (starting from the first planning application submission in 1999). In other words it offers a good insight into the workings of Berkeley's ‘development management’.

The 3.5 ha site, lies between the Battersea Power Station site and Battersea Park. It was created by joining Battersea Wharf, Spicer Cowan Wharf and the space underneath the railway arches that was lying between the two. It has a 300 m river frontage, is inside the Battersea Park Conservation Area and was a site with contamination hotspots dating back to the days when it was used by the railways and as a wharf. The three constituent sites were bought outright by Berkeley Homes in 1999. The owner was in receivership and this was the only route left open by the receivers (Price Waterhouse Coopers). This in turn meant that the developer was faced with the uncertainty of the Local Planning Authority refusing permission. However the early engagement with the LPA and the substantial know-how in planning negotiations allowed for this uncertainty to be managed.

The first proposals put forward in 1999 by Berkeley envisaged a ‘Corbusian’ layout for the site, as one interviewee put it. In any case, following consultation with the Local Planning Authority (London Borough of Wandsworth) and CABE the plans were revised to form a continuous urban frontage along the side of Battersea Park and along the railway lines (Picture 1). This was done in order to maintain the urban form and the ‘feel’ of the area and to provide more coherent public space at the central core of the scheme. This revision process resulted in a less iconic scheme but of equally high density, with an initial plot ratio of 2.3:1 reflecting in part the willingness of the Local Planning Authority to promote development in the Borough. Contamination was not viewed as an insurmountable obstacle in spite of the important hotspots that existed on the site, it was seen as a manageable risk.

Following this preliminary negotiation process, which involved the submission and subsequent withdrawal of a planning permission, Berkeley applied for planning permission again in 2000, using ‘double tracking’,^4^ for a mixed use development of 608 dwellings, a health club of 3500 m^2^, 8500 m^2^ of office space, 370 m^2^ of retail space and a 235 bed 4× hotel. The density of the proposed development stood at 173 dwellings per hectare, the buildings would reach 11 storeys and would have several basement levels. Although double tracking is usually confrontational, in this case it was not seen as such by the Local Planning Authority who, in the words of an interviewee, “understood” the rationale behind it, evidencing good rapport between planners and developers. The proposal was granted planning permission.

This understanding seems to have been put to the test in 2005 when Berkeley's proposal to change the mix of uses for the last phase of the development was opposed by the LPA mainly due to concerns that the development would lose most of its employment space in favour of housing. Eventually, Berkeley's application was approved on appeal. Other than that, objections to the development were limited throughout the period it took for the scheme to come to fruition since, as an interviewee said, “there was no local community to upset and have them object or make petitions”. Despite some criticism about the relationship of the development to the river there was a limited number of objection letters whenever Berkeley applied for permission to develop a phase.

A special business unit (Chelsea Bridge Wharf Ltd.) with an overdraft facility with Berkeley Homes and thus with the Berkeley Group was set up to develop the site. Chelsea Bridge Wharf Ltd. operates autonomously and has full control over the development whereas Berkeley Homes plays a key role in financing the development. The project is self-financed; the initial outlay is repaid through sales revenue. This practice limits the Group's capacity to expand production but implies commitment on behalf of the Berkley Group and depends crucially on central fiscal control in order to balance out the cash flows from various projects and create an overall positive return on the capital invested. The benefits of this system of financing, which depends on equity and forward selling whilst allows gearing only when there is increased certainty about future cash flows are reflected in the Berkeley Group's low gearing ratio (13.5% in 2003 and below 1% in 2008) which made it less vulnerable to interest rate fluctuations and to the credit crunch that ensued.

Following purchase, the same multidisciplinary team which also included people familiar with the commercial aspects and sales, works in close collaboration with planning and architecture consultants to apply for planning permission. Berkeley Homes was directly involved in the negotiations for permission and planning gain. After permission was granted Chelsea Bridge Wharf Ltd. (i.e. Berkeley) sought to change it on several occasions in order to fit market conditions better. At least 60 applications for all sorts of alterations, modifications or additions to the original submission, some of them quite substantial, were submitted during the decade that it took to complete the development. For example the development started with 608 units planned but this number later rose to 723, then reached 842 and eventually exceeded 880 units (Table 3).

The interviews revealed that Berkeley teams start working on a site by formulating a general idea of what the development should be like (size, etc.). Each team member argues on ‘what will not or will not work’ from their point of view. This way responsibility is shared between team members but also the various aspects of the development that could generate uncertainties at later stages are tackled. This inter-departmental approach covers all aspects of the project, an example is apartment layout which is constantly changed in this early stage until, in the words of an interviewee involved in the process, “it felt right”. The team that designed Chelsea Bridge Wharf realised that part of what they do would be tentative, in fact the notion of ‘getting it right from the start’ was doubted altogether. Everyone involved in a development team is encouraged to constantly look into and review projects instead of trying to fix something from the start (as the JCT Design & Build would require).

Several interviewees pointed out that what is sold in residential sales is not property but a lifestyle. High quality construction is therefore a priority but more importantly, emphasis is put on fulfilling buyers’ aspirations for a unique lifestyle captured in the ‘luxury inner city apartment’ ideal. Individuality and character are the most important features in that respect, satisfying substantial but latent customer demand (Barlow, 2000). The capacity to customise Chelsea Bridge Wharf is the outcome of a flexible design and construction process. The approach is very similar to the ‘Fasttrack’ construction method based on concurrent time scheduling and overlapping production elements. The big risk with this method comes from the effort required to coordinate the process, maintain quality and avoid or resolve conflicts. To mitigate this risk, the teams within Chelsea Bridge Wharf Ltd. bring together company experts and occasionally consultants and try to find how they can make the most of the site, the apartment, etc. This means that the site design is an iterative process that follows a learning curve.

This constant re-think of the scheme is a key aspect of the Berkeley approach to development. Because of the scale and the nature of the project at Chelsea Bridge Wharf the developer had to stay involved in it for at least a decade although this is a project that is average in size, for Berkeley's standards at least. The strength and the nature of future demand is probably the most important uncertainty factor in development. Usually developers try to create developments that will suit the nature of demand at the time the project is conceived. The original scheme therefore is a reflection of the developer's perception of ‘what will sell’. This perception might be accurate to various degrees. But it is almost impossible to successfully forecast demand at every stage for a project that takes the best part of a decade from inception to completion.

In such schemes, responsiveness to the changing nature and strength of demand is imperative. The development and consumption phases have to be articulated in a way that would ensure profitability or at least survival. Four factors can ensure this flexibility: appropriate phasing between and within elements, ability to quickly change the type of products offered, ability to manage this change profitably and a corporate culture that accepts change as a natural state of affairs.

**Fig. 5.4:**
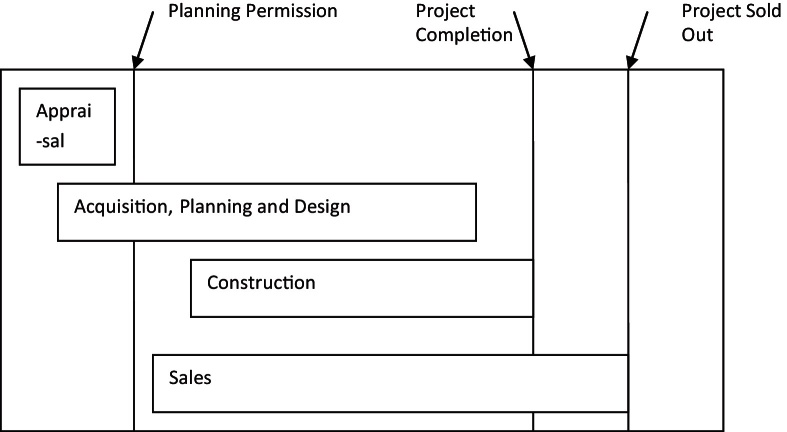
The interlocking stages of development in Chelsea Bridge Wharf.

This flexibility at the site level is a result of the flexible product development. As already discussed previously, the layout and design of the apartments are discussed in multidisciplinary design teams and are continuously re-worked “until it feels right” which implies a process that only ends when the last batch of apartments is constructed (Fig. 5.4). As a result the development is treated flexibly. This flexibility allowed Chelsea Bridge Wharf Ltd. to

• adjust the development's mix of uses during the development phase;
• ‘personalise’ the product (apartments).

Thus it was possible to sell a product bundle emphasising high quality, trendy, personalised, fashionable, stylish, ‘urban’ lifestyle. Interestingly the leasehold on a long lease ownership arrangement is another factor adding value to the development. The freehold for the whole development is sold to one investor who then becomes responsible for the maintenance of the whole development through a specialist agency. This in turn deals with a potential problem with the management of apartment block schemes where fragmented freehold ownership makes the maintenance of the building more complicated and in some cases impossible. This arrangement not only lifts that uncertainty, thus increasing the saleability of the properties, it is also an important factor in gaining planning permission and successfully negotiating the s.106 agreement.

Typically for development and construction methods based on concurrency, Berkeley's development approach requires very effective coordination and control mechanisms since the project is treated as a constantly evolving process which yields different outcomes as time progresses. Berkeley's approach is dealing with the uncertainties of development by increasing the quantity and flow of information both internally but also between the organisation and the external environment at all stages of development. Furthermore, it requires substantial coordination effort to allow these phases to overlap and thus benefit from the increased synergies that these overlaps allow. If successful, this approach maximises the ability of the company to respond to market fluctuations by modifying the mix of uses, the style, type and size of the apartments or the specifications of whatever space it is creating. At the same time, phasing allows parts of the development to be occupied whilst other parts are still under construction.

Chelsea Bridge Wharf is a characteristic mixed use high density development project similar in size and type to many others that Berkeley undertakes. Its approximately 900 units make it a big project by any standards but the Berkeley Group is involved in similar projects of 1200+ units, thus although it is a typical example in a uniquely marketable location it is by no means the biggest or most complex project currently undertaken by Berkeley. Having said this, Berkley itself recognises that their approach is not scalable and thus there are limits to the company's growth. These limits are imposed by organisational factors which determine a workable balance between devolution to autonomous teams and central management control and less by the intricacies of the development process itself.

### 5.2 Wimpey and Falcon Wharf

#### 5.2.1 The Wimpey approach

Wimpey followed a rather standard approach to development, trying to minimise turnover time. Profitability was based on the ability to secure suitable sites then get planning permission as quickly as possible, market and sell the houses and finally transform the income into new land purchase to start the cycle all over again. At the same time, strategic landbanking offered the company an edge over its competitors not only in securing a steady flow of land but also in profiting from land appreciation. Strategic land and strategic planning capabilities were given prime importance and were very well developed.

George Wimpey had, as Taylor Wimpey still has, its own network of land managers who were looking for sites and the group only partially depended on agents and land owners coming to them. Each business unit had control over development issues but strategic and financial issues were dealt with centrally.

The Group financed its operations through a combination of retained profits, bank loans, and long term loans in the form of US$ private placements with a group of US insurance companies. All loans were raised centrally by the Group's Treasury Department (George Wimpey Plc, 2001, p. 19).

Therefore, business units were able to analyse local circumstances quickly (planning conditions, UDP allocations, etc.) and rapidly locate and acquire or secure sites that satisfied a stringent set of conditions (well-located, etc.). The exception to this approach was George Wimpey City a unit specialising in ‘the high rise market’ which solely relied on offers because it was new and small and had not managed to establish the type of know-how and networks required for a proactive landbanking approach. The company was faced with teething problems and in 2006 the decision was taken for George Wimpey to exit ‘the high-rise market’.

The understanding of the development process as a process of adding value to land (and monetising as much as possible of it) is not uncommon in the housebuilding industry and the Wimpey managers who were interviewed shared that view. Interestingly however, at the same time that Berkeley was going down the regeneration route, a senior manager familiar with Wimpey's landbanking strategy felt that PDL in their landbanks was creating problems. In response to a question regarding Laing Homes (a company focused on high-end customers with 90% brownfield sites in their portfolio which Wimpey acquired in November 2002) an interviewee with good knowledge of Wimpey's practices said: ‘Their (i.e. Laing's) landbank is not entirely hopeless’.

In 2004 however, David Livingstone (Wimpey's Divisional MD for Laing) acknowledged in a presentation to visiting analysts that Laing's landbank contained sites at “better locations with larger plots” and that Laing was delivering a “premium product driven by local management” which focused on higher specification and high quality design (Livinsgtone, 2004). In his view Laing's equivalent in the automotive industry was Audi and Wimpey's equivalent was VW.

Although Laing's business model was not explored further as part of this research its focus sounds very similar to what Berkeley was focusing on at the same period, thus the way Wimpey managers saw Laing's practices highlights the differences between two different approaches to housebuilding. When it came to presenting the advantages from the merger, Livingstone pointed out that Laing had issues with controlling costs and therefore Laing should withdraw from high end markets. In his view Laing could benefit from “Margin improvement through ‘sweating’ overheads and build cost efficiency” and “benchmarking and build cost reduction programmes” whilst Wimpey could learn more from Laing's “land consultation and PR” capabilities. “Improving land purchase terms”, “working out poor historical sites” and “improving the land bank” through sharing large sites and “better cash management” where also mentioned as interim goals following the merger. What all this points to is the emphasis Wimpey placed on efficiency gains through asset sweating and cost control (competition for the present, see Section 2), considered by Wimpey to be a weak spot in the way Laing was doing things and something that Wimpey itself was very good at.

Wimpey decided to enter the PDL redevelopment market by establishing a new business unit (more about this in the section that follows). George Wimpey City, as the unit was eventually called, followed a different approach to that of Berkeley. In the words of one senior manager they did “what a true developer does”, they brought all the necessary elements together and they managed the process. They worked with contractors under JCT ‘Design and Build’ contracts which consequently meant that there was limited flexibility to alter the scheme once construction begun. This flexibility was further limited by the size of the developments which George Wimpey City was undertaking in the mid-2000s, up to 150 units. This size of development is equivalent to one big apartment block like Falcon Wharf, a flagship development of George Wimpey City. Although this size is 1/6–1/8 the size of developments like Chelsea Bridge Wharf it is still a significant endeavour, bearing in mind that the vast majority of UK housebuilders will produce around or less than 150 units a year.

The strategic review of 2005/2006 which led to a decision to mainstream PPG3 compliant schemes and withdraw from ‘high rise exceptionalism’ meant that most ‘high rise’ projects belonging to special units had either to be shelved or sold and eventually meant that the 800-unit ‘Green Bank’ project in Leeds had to be abandoned in 2007 although the first two phases had sold out. The capital intensive nature of these projects and the concern of the mother company that the apartment market was fragile led to the decision to exit the market altogether at a time when George Wimpey City was upscaling.

The effects of policy restrictions on land inputs and the type of response chosen had acute consequences for Wimpey. In mid-2000 the company initiated a long term strategy of diversifying their landbank to reduce the proportion of ‘short-term’ land (i.e. land at an advanced stage in the development pipeline) in favour of more ‘mid-term’ and ‘long term’ holdings. In the 2006 presentation of Peter Johnson (the Group CEO) to the AGM, the short term land bank is quoted as being faced with a “rapid unwinding of stock profits” (Johnson, 2006, p. 14). In the same presentation Johnson reiterated that the company was faced with adverse market conditions (especially a reduction in total transactions and a short order book) and was thus redoubling its efforts to diversify their product mix (mainly towards more affordable housing) whilst withdrawing from non-performing businesses like high-rise (i.e. the niche PDL brands mentioned in the previous paragraphs). Buying larger sites at better terms and cutting costs were to be key in driving future profitability. This approach however came late in the market cycle thus the company run into difficulties following the 2007 downturn in the UK. As a result it merged with Taylor Woodrow, a housebuilder with similar profile and size in a deal that gave 49% of the new company to previous Wimpey shareholders.

The company's approach appears to have changed thereafter and in 2010 the Taylor Wimpey annual report (Taylor Wimpey, 2010, p. 4) explains that the company creates and delivers value:

• By “purchasing the right sites, in the right locations at the right price”.
• By “designing a sustainable community that meets the needs of local residents, is attractive to potential customers and provides attractive returns for shareholders” through “a consultative and iterative process of community engagement”.
• By controlling cost and quality through careful procurement and subcontracting.
• By exceptional pre- and post-sales customer care.

Their process begins from a network of strategic land experts in the UK who are tasked with identifying areas where population growth, or other local demand, could create opportunities to promote land with no current planning consent through the planning system (Taylor Wimpey, 2010, p. 5).

The company claims that they “…have a strong track record of consultation with local residents prior to developing large scale communities.” and thus they

…are able to identify the best use of land to meet the needs of local residents, ensure that we have a mix of homes that meet market demand and that the site is optimised for safe, efficient and considerate development. (Taylor Wimpey, 2010, p. 5)

They explicitly mention that their experience on community consultation puts them in good stead in view of the provisions of the Localism Bill and its requirements.

Housebuilders like Wimpey faced (or believed they faced) the biggest bottlenecks in the planning application phase. A usual complaint of housebuilders and in this case of Wimpey's management teams is that local planning authorities are unresponsive to government policy in the sense that local councillors and planners do not easily approve of developments that adhere to the new higher density requirements and that they have ‘excessive’ demands from the s. 106 agreement. The technological barrier for traditional construction methods stands at 6–7 storeys which means that densities above a certain limit cannot be achieved unless site coverage increases and/or unit size decreases.

#### 5.2.2 Wimpey's adaptation to policy change

George Wimpey Plc, was one of the biggest UK housebuilders in terms of volume until 2007 when it merged with Taylor Woodrow. The key feature of Wimpey's strategy prior to 2007 was the focus on tried and tested recipes that have characterised their way of doing business for several years. Faced with the changes in government policy and in the wider business environment, the company tried to make the most out of existing know-how by slightly adapting it to the new circumstances while on the other hand they tentatively and rather belatedly explored ‘new ways’, based however on the tried and tested recipes with regard to development and market risks. Following the merger the new company, Taylor Wimpey, implemented a strategy which emphasised the importance of land in the value adding process and highlighted the significance of changes in policy and community engagement. However, the discussion in this part will mostly cover the years up to 2007 mainly because the merger itself is a liminal event for Wimpey.

During the 30 years preceding the merger with Taylor Woodrow the company shifted from a construction conglomerate with global reach to a housebuilder covering most of UK's housing markets both geographically and in terms of prices and products offered, with significant activity in North America and presence in Spain too. The company had a long history of Mergers and Acquisitions (M&As), a strong indication of ‘competition for the present’ (see Section 2). Indicatively, in 1995–1996 Wimpey exchanged assets with TARMAC in a deal that merged Mc Lain Homes (the housebuilding arm of TARMAC, a business almost as big as Wimpey Homes) with the Wimpey Group. In 2001 George Wimpey paid £461 million to acquire Mc Alpine Homes from Alfred Mc Alpine Plc that was then in a process of transforming from a construction group to a facilities management and utility services business (Mc Alpine, 2005). Consecutively, in 2002, Laing Homes was also acquired. This was a specialist PDL housebuilder who had developed a highly innovative design and construction skills base that allowed construction of housing “tailored to suit” each individual site (Laing Homes, 2005). Laing had a very strong track record of building Housing Association housing and upmarket PDL developments in London and the Home Counties.

These M&As were to some extend facilitated by borrowing, thus gearing in 2006 stood at 23% following a sustained effort to bring it down from 34% in 2005. This approach allowed George Wimpey Plc to extend its geographical coverage and product and price range to cover most of the UK. Among other reasons, the company was using diversification into various market segments and geographical areas as a risk management strategy. The 2006 Annual Report (George Wimpey Plc, 2006, p. 2) summarised this approach:

Our 26 regional businesses and three satellites in the UK give our operations significant scale and truly national geographic coverage. Each business unit provides a range of products, from one bedroom apartments and starter homes to large detached family homes.

This approach to housebuilding was in striking contrast to the Berkeley's strategic orientation and hints at a different attitude towards risk and uncertainty, much closer to the traditional housebuilding strategies of diversification into many market segments, wide geographical coverage and reliance on M&As to acquire skills and increase market share.

Wimpey went through several reorganisations in the years prior to 2007. Indicatively the 2001 reorganisation aimed at reducing costs (staff, building/procurement) and to increase long term performance. It led to the decision to rebrand all the products of the different divisions to ‘George Wimpey’. Other aims were:

• to improve the landbank, partially achieved through the acquisition of Laing;
• to reduce regional businesses from 29 to 21;
• to devolve responsibilities from the Group to the divisions;
• to establish 2 companies focusing on inner city developments one for inner London and one for other major UK cities, based in Manchester);
• To expand the presence to the US market.

These aims were essentially consolidating the direction that the company had taken in previous years in an attempt to tackle the main challenges facing big non-PDL housebuilders in an era of transition to new development types. The 2001 Annual Report illustrated a point also established through interviews and the analysis of the company accounts. Wimpey was adjusting to PDL redevelopment and this was affecting the types of dwellings produced. At the same time this delay in changing the product mix is telling of the difficulties Wimpey was faced with, as the following quote indicates (George Wimpey, 2001, p. 15).

The product range now available to customers is well spread both in geographic and demographic terms and is well positioned to cater for a broad spectrum of the market. Inner-city and bespoke developments have continued to expand as dedicated management focus on this area of the product portfolio. The level of activity on brownfield sites has grown to 45%. Apartments now represent 11% of completions and 58.0% of homes completed in the year were detached. With a growing shortage of skilled labour, reassessing how homes are constructed whilst maintaining a high level of customer satisfaction is becoming a greater priority. A Research and Development Manager has been appointed who will concentrate on the research aspect of the challenge during 2002. This will entail assessing already proven building techniques around the world to ensure the Company is well prepared for changes over the next generation of housebuilding.

It is worth noting that while the company was moving towards more flat production, it still produced a substantial number of detached houses. Following the acquisition of McAlpine (completed October 2001, £463 million) Wimpey not only increased its presence in the booming south but also acquired a big landbank of high quality that would cover the Group's needs in the south for two years after the acquisition (George Wimpey, 2001, pp. 5, 14). The landbank was further enhanced in terms of PDL following the acquisition of Laing yet the composition of the company's output changed very slowly.

Increased operational efficiency and streamlining was an expressed goal in the corporate strategy documentation whereas themes like urban regeneration and sustainability first featured in the company's strategic visioning well into the 2000s. Peter Johnson (the company's Chief Executive) succinctly summarised the situation in 2006:

In 2001 we restructured the business to one with an efficient overhead structure and product range and low build costs. We acquired McAlpine Homes and invested in our landbank, raising hurdle rates to deliver improved margins. We acquired Laing Homes to give us a second brand to support organic growth. However, our landbank remained short and our strategic land limited. With few sites coming forward with older planning permissions, the implementation of Government planning policy, PPG3, impacted us faster than most. Underlying build costs rose sharply as our build efficiencies were eroded. When prices stopped rising, margins fell as high build costs on bespoke PPG3 schemes compounded the higher land costs associated with a short landbank. Lessons have been learned, and changes made. Going forward, all regional businesses will have the opportunity to use both the George Wimpey and Laing Homes brands. This will allow better use of large sites and provide greater returns on future land purchases. We have re-established an efficient overhead structure and product range which meet the needs of today's market and planning regime and which again give us lower build costs. The use of standard building elements and a full range of PPG3 preferred house types are re-establishing our former time and cost advantages, whilst enabling us to meet local planning requirements for variety of elevation without the requirement for bespoke developments (George Wimpey, 2006, p .6).

Until that point, Wimpey's senior management appeared to be reluctant to radically reorient the company in response to the new post-1995 policy regime and the business environment it created. Instead it seems to have followed the model which Ball also refers to in his work (Ball, 1983). This model is based on spreading market risk by covering all the UK and several market segments then hedging that risk by operating overseas, i.e. in the US market and to a lesser extent in the Spanish market. Well into the 2000s (in 2006) the company referred to its activities as housebuilding, with little mention of regeneration and the corporate website scarcely mentioned issues of sustainability and the latest government agenda. PDL redevelopment was treated as a market niche which was to be exploited by specialised subsidiaries operating in high margin areas like the Thames waterfront.

Parallel to the effort to increase market share and skills base through M&As and as part of the strategy to expand into new markets by establishing new business units, Wimpey decided to move into what it perceived as the growing niche market for inner city apartments in high density developments, like multi storey blocks. The company realised with some delay that there existed an emerging market niche for upmarket apartments on prime locations in London (mainly riverside) or other major cities and that the skills required to build high rise PDL developments are specialised and new to housebuilders. Indeed, more common methods (like timber frame) are economically and technically feasible for buildings up to six to seven storeys. Above that height new materials and construction methods have to be deployed (for example structural concrete, curtain walling) which are unusual for the traditional housebuilding industry but are widely used in commercial property construction.

Following the acquisition of Alfred McAlpine and thus the acquisition of its portfolio of ‘urban projects’, two business units were established in 2001, George Wimpey City to cover the metropolitan areas outside London (mainly covering Manchester and the North) and George Wimpey Central London for the London market. The two units merged during the first months of 2004 but the merged unit maintained offices in Manchester. George Wimpey City was the only unit that had the capacity and skills to build high rise apartment blocks by outsourcing most of the necessary skills. It deployed these skills to tap into the luxury end of the market, thus directly competing with the Berkeley Group. Although Wimpey established Inner London special teams in 2001, product composition has been changing towards more flats since 1999, indicating a product shift even before the new specialised business units begun to have an influence but four years after the government announced its PDL redevelopment targets.

The business unit had a very narrow remit: to develop high rise apartment buildings in inner city PDL sites and was not initially involved with complex long term projects. Many of the sites had a social housing element and most of them were mixed use. Although they were not the only units of the George Wimpey group that worked with PDL sites they were the ones who specialised in multi-storey apartment buildings. The other units in the group were developing more conventional housing schemes with traditional methods thus tapping into lower margin markets of low density PDL and medium density PDL or into the lucrative but restricted low density non-PDL.

Wimpey treated PDL development as a new market niche in to which they expanded by establishing new business units. As a result in 2005 the group produced 66% of its output on PDL and still maintained 34% non-PDL production (in comparison the equivalent percentage for Berkeley was consistently over 95% since 2001). This strategy however, regardless of its advantages or disadvantages, attracted a ‘UTF follower’ characterisation from the GLA and eventually this more conservative approach turned out to have limited long term benefits, as discussed already.

The new entity's (Taylor Wimpey's) approach to generating value is rather different and puts emphasis on the role of land in the process as described in their 2010 annual report (p. 4):

We generate value for our shareholders through managing our investment portfolio of land to deliver optimal returns. These returns are created through identifying the best opportunities, adding value through the planning process and designing places to live that meet the local demand. We deliver this value through safe, efficient and considerate development of these communities and helping our customers to buy and move into their new homes.

In the same document it becomes clear how value added is derived at:

we have a team of strategic land experts in the UK who are tasked with identifying areas where population growth, or other local demand, could create opportunities to promote land with no current planning consent through the planning system. (p. 5)

and

Rather than seeing homebuilding as the driver of value, we see it as the way to deliver the value that we have created through selecting land and optimising its value. (p. 9)

This is a very strong hint at the importance of land speculation, rather than housebuilding itself, in driving the strategies of a housebuilding company.

#### 5.2.3 Wimpey's performance

**Fig. 5.5:**
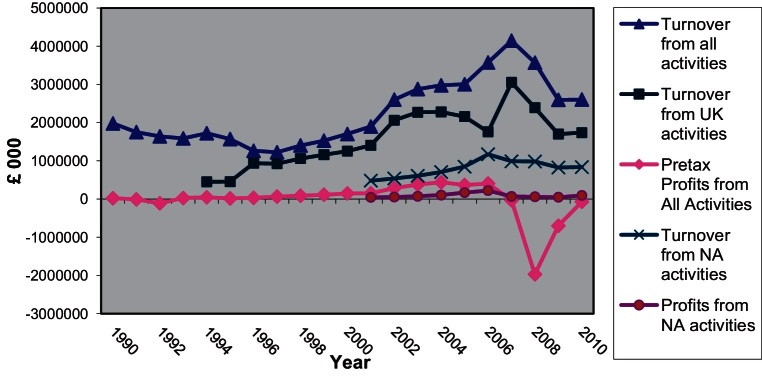
Wimpey, annual turnover and profits 1990–2010.

*Source*: Selected company annual reports 1990–2010.

**Fig. 5.6:**
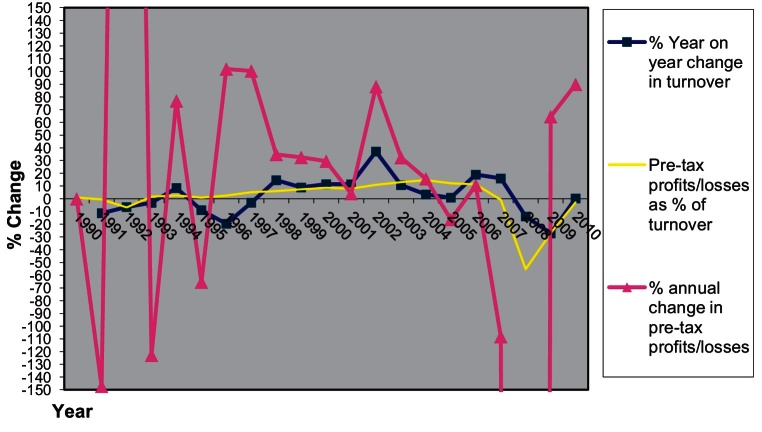
Wimpey, nominal changes in turnover, profits and profit margin 1991–2010.

*Source*: Selected company annual reports 1990–2010.

Wimpey was using geographical diversification globally as a way to manage market uncertainty. Its declining performance in the UK market sped up after 2003 however the US business picked up substantially during the same period, acting as a buffer to what otherwise would have been a significant drop in turnover and profitability. Indicatively, the average sales price in the UK dropped to £178,000 in 2005 from £185,000 in 2004 and profit margins declined to 12.9%. During that same period, US average sales price rose by 7.6% to $313,000 and the profit margin rose to 20%. The year after that, 2006, saw a further drop in the UK average sales price effectively squeezing margins and turnover (Figs. 5.5 and 5.6).

It is important to note once more the significance of the US business activity which for most of the 2000s stood in sharp contrast to what was happening in the UK operations. Dwelling output, nominal turnover and profits for the US business arm were steadily increasing since 2000 when data is available. The slowdown that the US market witnessed after 2005 following the significant increases in the Federal Reserve interest rates at the time did have an impact though. The company's chairman summarised the situation as follows (George Wimpey, 2006, p. 6):

The housing market in the UK remained difficult throughout the year, with the total number of housing transactions for 2005 17% below the previous year. Against this background, our businesses did well to deliver total UK volumes similar to last year as well as significantly increasing our forward order position entering 2006. The increased use of incentives needed to achieve this, along with the impact of a shorter landbank, resulted in reduced operating profits and margins. By contrast the US housing markets in which we operate remained very strong, with national housing starts reaching record levels. We continued to push forward strongly with our growth plans and delivered higher volumes, margins and operating profits. In the US too, we have come into the new year with a far stronger order book.

**Fig. 5.7:**
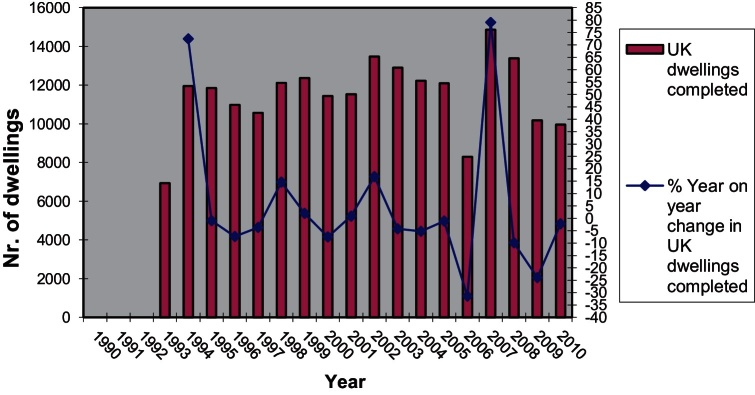
Wimpey, nr. of UK units completed and year on year change 1993–2010.

*Source*: Selected company annual reports 1990–2010.

A complete dataset on units sold by Wimpey in the UK is difficult to find, data on units completed show that after the asset exchange with TARMAC the number remained practically stable at around 12,000 units (see Fig. 5.7). The acquisition of two significant housebuilders, McAlpine and Laing Homes had a minimal effect on output which dropped dramatically in 2006 reflecting what the 2005 annual report called “*…*the most challenging housing market in the UK for a number of years” (George Wimpey Plc, 2005, p. 2). After the merger with Taylor Woodrow output rose initially but then stabilised to lower levels as well. Prior to the merger the two companies were building around 8000 homes each in the UK, in 2010 their combined UK output was 9962 units. The benefits of the merger therefore came from shedding excess capacity, staff layoffs, procurement, etc.

**Fig. 5.8:**
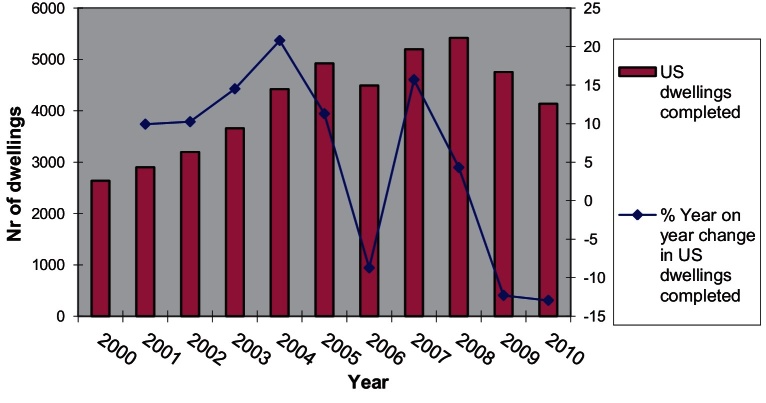
George Wimpey (and Taylor Wimpey), nr. of US dwellings completed and year on year change 2000–2010.

*Source*: Selected company annual reports 1990–2010.

Both companies had significant US presence although following their merger a lot of capacity was scrapped both in the US and in the UK. Thus, post-merger US output was more or less the same as the output of each company separately pre-merger (see Fig. 5.8). Finally, the US business of the merged entity (Taylor Wimpey) was sold in mid-2011.

The course of Wimpey's profitability both pre and post-merger is strikingly different to that of Berkeley. Under the sustained pressure of government policy on land input the firm's product mix slowly shifted towards flats whereas output volume remained steady. In effect Wimpey found itself selling more of a type of dwellings with a potentially lower average selling price per unit while it was stripped of the capacity to sell expensive dwellings on greenfield land (detached housing, etc.) and was not able to profitably enter the high end apartment market. While this dynamic was underway the primary source of turnover increases for George Wimpey was the general uplift in housing prices. Company profitability benefited from a sustained effort to increase efficiency (a recurring theme in George Wimpey's strategies with ambivalent outcomes), geographical diversification into the US and Spanish market, as well as some major reorganisation efforts combined with M&As.

Thus throughout the period examined in this paper, the focus of the company was hardly, if ever, on future market share in regeneration and creating sustainable communities were not at the core of the company's strategy and thus were not affecting its business model substantially until 2006. It is indicative that as part of its 2006 reorganisation the company moved out of units specialised in high-rise (i.e. high density PDL like Falcon Wharf) but at the same time tried to mainstream and standardise PPG3 compliant development. However, another strategy appeared to be reaping rewards. In 2005 the company's sales of affordable housing went up by 48% (average selling price at £98,600, up 10%) whereas private dwelling sales in the UK dropped by 5% (average selling price £188,600, down 2%).

It was unfortunate for the company that almost as soon as this reorientation begun to bear fruit (in 2006 UK turnover was up 11% operating profit was up 14%) the market deteriorated dramatically. The simultaneous difficulties in all 3 major markets that the Group was involved in (UK, US, Spain) eventually took their toll and undermined the position of the whole Group. In July 2007 George Wimpey merged with Taylor Woodrow in a deal which saw Taylor Woodrow shareholders getting 51% of the shares in the new entity.

#### 5.2.4 Wimpey in action: ‘Falcon Wharf’

The 0.496 ha site is bounded to the west by the Thames, to the east by Lombard Rd., to the south by the Heliport and a timber merchant's warehouse and to the north by another residential development (Oyster Wharf). The site was under Council ownership and was used as a vehicle repair depot, building works depot, highways depot and laundry. At the end of the 90s Frogmore Estates, a property investor/trader bought it through their subsidiary, Harbour Land Ltd., with the intent of getting planning permission and either sell on or find a partner for a joint venture. This was in line with their standard business practice, especially for housebuilding where development activity is usually undertaken by a development partner. In the case of Falcon Wharf the site was sold with detailed planning permission to Wimpey who implemented it with slight alterations as will be discussed below. Following their strategic review of 2005/06 Wimpey set up a joint venture called Falcon Wharf Ltd. with the Royal Bank of Scotland and sold to it the land and associated development works for £31.7 million.

In similar fashion to Chelsea Bridge Wharf this site was also partially in use prior to its acquisition by Frogmore and various applications for planning permission had previously faced difficulties with planning conditions or the s.106 agreement. Falcon Wharf (Picture 2), has a plot ratio of 4:1 and the building was to be 55 m high (later revised to 59 m). This site is also on a prime location, situated within a fabric of active warehousing and light industrial uses that are now enmeshed with new luxury residential developments. In this particular case, a heliport is also present and as any visitor to the area can say, it is also very intensively used. The site was originally owned by the London Borough of Wandsworth and is located on a strip that witnessed a lot of speculative activity during the last 15 years. Most of the waterfront to the north and south of the site has been or is currently developed for residential/mixed use schemes. It was however hardly a ‘brownfield’ site as it was actively used by more than one users for more than one uses.

Originally, Harbour Land Ltd envisaged a development with 119 dwellings, 14 of which (12%) would be affordable, a hotel with swimming pool, a small restaurant, 207 parking spaces and a riverwalk extending over the water which would render the dock unusable. Following consultation with the LPA the scheme increased in height and size but alterations occurred in its relationship to the river as well. The riverwalk was redesigned to be built over land, the dock remained usable and became better integrated into the site. It applied for planning permission in 2000 for a 149 unit scheme to include a restaurant and office space as an alternative to the hotel. Wimpey bought the site from Harbour Land with planning permission in 2002 and applied for alterations to the permission in order to make the development more profitable by tuning it better the market conditions prevailing at the time. This new application was faced with significant difficulties and delays. This was because the affordable housing policy requirement had changed between the date planning permission was granted to Frogmore and the date Wimpey applied for the alterations. Finally, the developer and the LPA agreed that affordable housing would be provided off site but this only happened after the application attracted the attention of the Mayor of London. Construction, based on those alterations, begun in 2003 yet planning permission was granted one year before completion, in 2005, due to problems with the section 106 agreement. After 2006 the joint venture scheme (Falcon Wharf Ltd.) applied for some alterations mainly with a view to expanding the size of a few top floor rooms and of the penthouse (apparently bought by Iqbal Latif). In 2007 Iqbal Latif bought the freehold of the whole scheme and reinstated the hotel, a change which required the provision of a gym and spa as well as changes in the restaurant space. After it was completed in 2006 and even more so after the hotel was developed the scheme attracted a lot of attention for its high architectural and environmental sustainability standards and received several awards (Table 4).

There may not have been a significant local residential community in the area in 2000 but there was a significant number of small business interests. From the 33 neighbours only three objections were received originally, plus two more after the final revisions. The Heliport mounted the most vociferous opposition to the development mainly because of concerns about the effects that a high rise tower would have on air navigation and landing procedures because of the changes in wind circulation. After the involvement of the new owners in 2007 the number of planning applications increased (to reach 17 in total by 2010), partially reflecting changes in the mix of uses and the composition of units to align the development with the market situation and partially reflecting cosmetic and design alterations to the landscaping and the building. At that stage, there is also a more established local community so the number of participants in the planning consultation also rose though often the representations were in favour of the proposed changes.

If one was to apply de Magalhaes’ ‘Functions and phases’ matrix in the case of this development one would find that the most interesting feature in terms of the way the different functions and phases are articulated is the separation between the phases of Mediation and Development (see Table 5). Indeed Harbour Land handled the mediation phase and profited from it but this separation into discrete stages allowed George Wimpey City to be involved in the Development phase in terms of ownership and finance provision with the architects, Burland TM, bridging the gap between mediation and development.

All other aspects of the development process were handled by agents external to George Wimpey City. This indicates that in essence George Wimpey City acted as a developer who allocated a certain amount of resources on their project and outsourced most other functions in an effort to make a return as quickly as possible based on a relatively fixed development proposal. This is in contrast to the direct involvement of Berkeley in most phases and functions, constantly striving to add value to the development by a series of modifications and alterations.

The functions phases and interactions shown in Table 5 are as follows: (1) freehold sale; (2) internal capital financing; (3) asset purchase from RBS; (4) consultancy arrangements; (5) planning or other permissions; (6) JCT Design & Build contracts; (6) bank financing or other sources suitable for small investors or owner occupiers; (7) rental agreements. Prior to the formation of the joint venture, George Wimpey City had an overdraft facility with the parent company (George Wimpey Plc) similar to the arrangement Berkeley Homes/Chelsea Bridge Wharf and indeed similar to the arrangements of most housebuilders who rarely if ever use development specific borrowing facilities.

The project was self-financed, the original capital inflow from the parent company was to be repaid sales begun. However, the timescale of the development was such that this turnover period was planned to be rather short and forward sales would ensure that the negative balance was limited even further. In reality, the problems with the planning permission meant that sales could not proceed as fast as it was envisaged, causing difficulties with the cash flow of the scheme and thus exposing the parent company to risks to which it did not want to be exposed. The lesson was learned and forward selling was employed more successfully in later developments.

Until Falcon Wharf Ltd. was established, George Wimpey City operated autonomously and had full control over the development. They started work on a site either by formulating a general idea of what the development should be by doing a preliminary appraisal. They did not have the capacity to locate land through their own agents so they relied on sites coming forward through third parties (agents or developers). The site was then appraised by the Development Manager in cooperation with the cost consultant and if the appraisal showed that it was feasible then they either bought an option or bought the site outright depending on the circumstances. The site for Falcon Wharf was bought outright since it was offered with planning permission following the completion of ‘Mediation’ by a third party whereas several other sites were inherited from Alfred McAlpine at similar or more advanced stages.

**Fig. 5.9:**
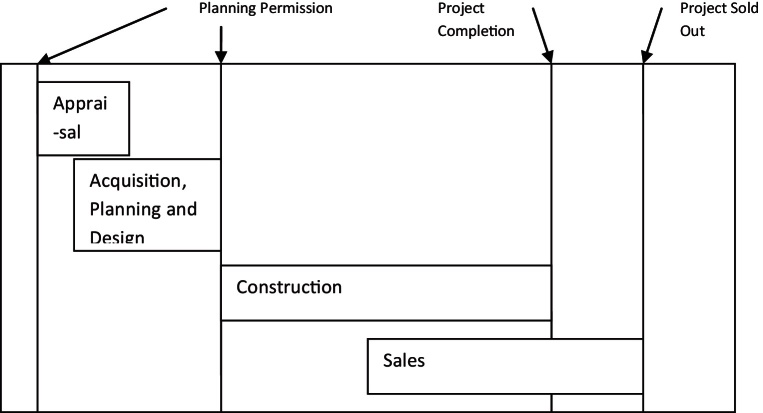
The distinct stages of development in Falcon Wharf.

A team comprising the Development Manager, planning, architecture and cost consultants revised the plan and sought to change it to fit market conditions better. However, although the changes were minimal, changing the s. 106 agreement in order to provide affordable housing was difficult. The process is relatively linear and separated in discrete stages essentially under the control of one person at any one time, the Development Manager during the planning stage and the Commercial Director during the development phase. This compartmentalisation of the development process is an important aspect of the way Wimpey treats development (Fig. 5.9).

The Development Manager organised the process and made key decisions about the site, sometimes in cooperation with other departments up to the point where development begun. Thereafter the Development Manager handed over to the Commercial Director who had responsibility over sales and the Project Director who had responsibility over construction.

Since this particular site was brought to Wimpey with planning permission through personal contacts (between Carillion, Frogmore/Harbour Land and Wimpey) the profit potential was lower because the added value of gaining planning permission was included in the price of land. The design for the site was re-worked with the original architect's help in order to maximise development potential, this again was the usual GWC approach for sites which were bought with planning permission. This particular site however was given special attention since it is a prime waterfront site and therefore GWC identified its potential to be a flagship project. To achieve those characteristics the product had to undergo some modifications that would make it more attractive to the potential clients who would aspire to the lifestyle the development represents. The uses originally envisaged by Harbour Land also had to be changed, the content of the commercial element in particular. Therefore the Hotel gave its place to a bigger restaurant and a bigger office element only for the decision to be reversed after the market was tested and no tenants could be found for the office space.

The marketing strategy for this development also depended on selling a ‘lifestyle’, capitalising on the vibrancy and vitality of nearby Battersea, a message that was reinforced after the hotel–restaurant–spa complex where put in place. The development even had its own website which amongst other things included a list of upmarket restaurants in the vicinity of the development.

Within GWC the Development Manager acted as the coordinator of the process, bringing together the company experts and occasionally the consultants in order to find how they can make the most of the site. Due to the restrictions imposed by the existing design, the short timeframe, the nature of the D&B contractual arrangement and the compartmentalisation of the development process this attempt did not result in radical transformations other than the change in the commercial element. Changes of the same magnitude and of the same nature as these that took place in Chelsea Bridge Wharf were impossible because of the procurement method (Design & Build) which is less flexible in terms of permissible quantitative and qualitative changes in the development once construction has begun. There was also limited possibility for customisation based on customer feedback and forward sales. This flexibility however is not seen as necessary in this approach to development since the development is relatively small and has to finish in one phase, as soon as possible after starting.

This approach, emphasising speed of delivery of the whole development in one phase has another interesting implication. If this approach was to be followed in big mixed use development schemes then they would need to be developed in one phase from start to finish. As the size of the development gets bigger the uncertainties and risks surrounding all aspects of the development increase. Thus, although it might be technically possible to produce a development of 900 units in one go, provided that enough capital is available, the market risk inherent in successfully developing and selling so many units in a short period of time makes it an endeavour difficult to undertake without substantial financial backing. As the interviews confirmed, George Wimpey City started off with developments sized between 50 and 70 units and gradually moved to developments up to 150 units, then eventually upscaled to 800 units. This size however proved to be unsuitable for the ‘risk profile’ that the parent company saw as manageable.

The joint venture with RBS not only boosted the profits of the parent company but also allowed it to spread the financial risk of the development. A similar joint venture with Barclays Bank was set up for another similar development, GN Tower. However, it was not possible to explore further the implications that this move had for the development process model that Wimpey applied to PDL projects since the company merged with Taylor Woodrow. It is interesting however that the latest owner of the freehold (Iqbal Latif) spotted the opportunity presented by the empty office space, the building's plant and the large restaurant, thus merged them into a hotel, spa and restaurant complex.

### 5.3 A second look into strategies, norms and competitive advantage

There are 10 key points structuring the theoretical background of this research, reviewed below:

(i) Changes in the business environment affect businesses in a fundamental way.
(ii) Economic, institutional and social factors act as filters that sort out which paths are to be followed from a multitude of feasible futures.
(iii) The capacity of a firm to survive a change in its environment depends on the fitness of its strategies and their translation to organisational routines.
(iv) A company's competitive strategy effectively relies on combining activities in a way that gives it an advantageous position against its rivals.
(v) If ‘new ways of doing things’ offer a competitive advantage then they are likely to spread via imitation or via the better chances of survival of the firms who have that advantage.
(vi) Economic actors are purposeful profit seekers but follow norms and routines in the pursuit of their goals, these norms and routines bind actors but are subject to reviews depending on the circumstances.
(vii) Strategic competition is about the future, concerns for operational efficiency and market share are about the present.
(viii) Strategic competition requires strategic intent which in turn requires an unorthodox, contrarian stance.
(ix) The development process is rife in risks and uncertainties at all its stages.
(x) Housebuilding has a wholesale and a construction aspect to it, the wholesale aspect is much more uncertain but has much higher returns.

The two firms examined in this paper treat housebuilding in very different ways, although they are faced with the same business conditions. The housing market is notoriously volatile and therefore notoriously difficult to predict, housebuilding is rife with uncertainty as became evident from the previous sections. This uncertainty and the brave assumptions that housebuilders have to make about the future course of the market has been the main source of corporate closures in times of sudden market decline.

The stakes were high for George Wimpey Plc and for the Berkeley Group from the moment that the sustainability agenda entered the mainstream of UK policy making. Within the social, economic and cultural context of the UK a restriction on greenfield development and push towards the brownfields was bound to raise a wide range of responses from absolute rejection to wholehearted support. A multitude of feasible future ways of producing the built environment opened up the moment the government begun to set targets on land reuse, densification and use mix. At least six of these scenarios have been identified in this paper. These potential new futures were more than mere possibilities, all of them were tried to a small or large extent by housebuilders around the country. Someone in the housebuilding world however would have to imagine what these future urban quarters would comprise, understand what it would take to get there and relate to the workings, the strengths and the weaknesses of their firm. Only few of these scenarios where viable, as companies like Persimmon discovered when they tried to develop low density PDL schemes.

Wimpey followed what seemed to be a conservative approach, realigning its strategic direction very slowly, apparently held back by the inflexibility of managerial mental frames and huge legacy costs mainly related to their landbank. It may well have been the case that they thought PDL redevelopment would be a temporary phenomenon or that it would not catch up given the cultural significance of the rural idyll in the UK. On the other hand Berkeley shed its old identity of high end greenfield housebuilder and engaged actively in a process of refining the skills and routines required in order to tackle the task of creating upmarket, high density, mixed use schemes.

Both companies developed strategies and employed norms and routines to tackle the risks and uncertainties embedded in the development process. At the macro level Wimpey continued to diversify geographically and to cover several market niches. It remained a company very much concerned about operational efficiency, speedy site turnover, standardisation and land speculation. It was buoyed by the upturn in the overseas markets it had expanded to but in the UK it stagnated in spite of rampant house price inflation. It tried to maintain or gain market share by M&As which however increased their gearing ratio substantially.

Berkeley opted for focus, both geographically and in terms of market coverage. With time it transformed itself to the dominant market actor in housebuilding in London. It has achieved this through a learning process, by testing the market early on and by strategically pre-empting the policies of the government. Wimpey went through a learning process as well, ‘lessons were learned’ said the annual report in 2006. Yet the realisation that the ‘genetic coding’ of the firm needs to change and therefore that the firm needs to reorient itself towards more profitable activities (like affordable housing and PDL dwellings) came too late to make a difference to Wimpey's fate.

At the project level, Wimpey established special business units to deal with the issue of PDL development although it had acquired two companies that had such expertise. George Wimpey City with Falcon Wharf was tapping into the market for high-end dwellings in mixed use developments for consumers seeking a luxurious lifestyle. Its marketing pitch was very similar to that of Berkeley. The process of development was treated in discrete stages with minimal overlap and limited information feedback loops. George Wimpy City for example had only limited involvement in the Mediation and Consumption stages. Emphasis was put on minimising exposure to uncertainty and risk within each stage by pursuing the fast delivery of the outputs of each stage so that the next stage could proceed. This latter attitude was the cause of conflict with the LPA and put the development at risk since it essentially went ahead without a finalised s.106 agreement. Not much room for collaboration was left after that.

Berkeley developed and mainstreamed throughout the organisation sets of rules, practices, norms, routines to manage uncertainty through a flexible approach towards development. This approach, which enhanced the firm's competitive advantage, is based on collaboration and on flexibility towards the planning and design of the product and relies on the incorporation of market feedback into the development process. If successful, this approach, maximises the ability of the company to respond to market fluctuations by modifying the mix of uses, the style, type and size of the apartments or the specifications of whatever space it is creating. At the same time, phasing allows parts of the development to be occupied whilst other parts are still under construction.

The success of the development project if this approach is followed depends more on the successful management and coordination of the project and much less on market conditions or fluctuations in the business environment. However, the practice of modifying a project from phase to phase in order to suit market conditions better might actually create an issue of community participation for the surrounding residents and users who were consulted at the early days of the project. The same applies for the tenants who move in at later stages and for the LPA who may discover that the end project will not be what they expected or wanted it to be.

The outcomes of these two approaches could not be more divergent. PDL redevelopment and urban regeneration where not mainstreamed at Wimpey and when the company was struck by a secular downturn in all the markets they had diversified to, they faced severe financial difficulties. Berkeley grew at a dramatic pace, its profit margins steadily remained the industry average or higher. The growth of the group is ‘organic’, based on tapping early on into hitherto unexploited market niches. A recent reorganisation exemplifies this, the Group's structure includes a division specialising in contemporary forms of non-market housing and a division keeping an eye on strategic land opportunities with some knowledge of lower density suburban housebuilding. Ironically, these are competencies that Wimpey also had and actually intended to develop further before it was struck by the turn in the market.

The comparison of the way these two projects were developed revealed quite a few similarities but also uncovered some key differences between the two companies. Berkeley, in the form of Chelsea Bridge Wharf Ltd. has applied a development method that capitalises on change by implementing an approach to development that emphasises information flow within the company and between the company and its environment. The result is that the project at Chelsea Bridge Wharf has undergone significant alterations throughout its lifetime and each phase of its development is much better tuned to the needs of the market, thus it can sell quicker and at a premium. Even when the dwellings are sold on and therefore the developer disengages, provisions are made that the scheme remains under a united Freehold therefore putting in place the conditions for a high standard property maintenance and management service.

This flexible approach does not mean lack of involvement as the process of mediation and development remain under the strict control of the company in its various guises. The scale of the whole development is also much larger than Falcon Wharf since the project is not treated as a ‘one-off’ but as a constantly evolving process which yields different outcomes at each phase. Chelsea Bridge Wharf is a characteristic mixed use high density development project like many others that the Group undertakes, as a matter of fact its size is significantly smaller compared to other projects which can be twice as big.

It is not possible to say with certainty whether Berkeley is a ‘contrarian’ company whose people possess an unorthodox way of thinking. It clearly has strategic intent and given the way the housebuilding industry operates and the criticism it regularly receives with regard to its pace of innovation it may well be the case that what Berkeley does, appears to be radical because a lot of its competitors follow more traditionalist approaches. Whatever the case may be however, the company enjoys a clear competitive advantage in the markets it operates and it dominates them to the point that it poses a threat to the competitive function of the London market for new-built housing and therefore to the land market as well.

## 6 Conclusions

Housebuilding in the UK has undergone an era of dramatic change in many respects since the mid-1990s. The main focus of this paper was the transformation occurring in housebuilding as a result of policy changes generating new risks and uncertainties for housebuilding firms. These changes promoted urban living and new types of urban environments, in the form of denser mixed use developments, more stringent environmental standards and a greater social mix. Arguably, the change of government in 2010 created some uncertainty as to the future direction of urban policy, the NPPF continues to focus on the re-use of PDL and the Localism Act embraces pre-application consultation.

Although it emerged as a concept in the early 1970s, sustainability has increasingly influenced British urban policy from the early 1990s onwards. Thereafter, UK governments started to favour city revitalisation and the return to urban living as opposed to suburban or rural lifestyles. These new ideals for urban living have been consolidated through a series of documents the most important of which arguably was ‘Sustainable Communities: Building for the Future’ (ODPM, 2003a). This document epitomised the Labour government's approach and advanced its implementation, either by establishing a set of principles that should guide future growth or by addressing the funding issues that accompanied this effort.

The brownfields vs. greenfields debate and the promotion of high density mixed use schemes in order to protect the countryside have had a significant impact on the housebuilding industry. That industry comprises firms whose business is the transformation of space from one configuration of uses and users to another. From a ‘production process’ point of view this is an industry whose ‘raw material’ is land (previously used or unused) and its output is dwellings or groups of dwellings. If one wants to affect change at the level of industry therefore, one very effective way to do it is to affect the input of land.

Following the detailed examination of the strategy-making process and the effects that each firm's approach had on its financial position and its output it can confidently be argued that there are significant differences between the way the two companies handled the development process and associated risks. Berkeley benefits from specialisation but is more exposed to one market in terms of geography and market segment. Wimpey on the other hand remained at the top of the league through M&As and was much more diversified.

The two companies examined in this paper treated housebuilding in different ways, although they were faced with similar business conditions. The housing market is notoriously volatile and therefore notoriously difficult to predict but the development process as such is not very certain either as Byrne (1996) has discussed.

A traditional ‘current trader’ (see DCLG, 2007) housebuilder response to this type of market has been to diversify in terms of geography and market segments. George Wimpey entered the 1980s as a construction conglomerate, active in most construction activities and ended the 2000s in a merger with Taylor Woodrow, one of the oldest housebuilding companies. George Wimpey was a construction conglomerate but eventually a radical restructuring effort led to an asset exchange with TARMAC in the mid 90s. Thereafter, George Wimpey Plc became almost exclusively focused on housebuilding and made sustained efforts to increase profits mainly by focusing on operational efficiency, a sign of ‘competition for the present’.

To complement this efficiency drive, the company expanded its geographical coverage and product range mainly through M&As. It also expanded its activity in the US market as a further way of diversifying its business and its market exposure. Indeed the US activity boosted the profitability and the margins of the group (20% operating margin in the US for 2005 compared to 12.9% for the UK). In the UK market the margins were close to the industry average and growth in terms of output was static, indicating that turnover and profit growth depended on housing price inflation, M&As and efficiency gains.

The involvement of the group into the redevelopment of high density PDL followed the same logic. The company found it difficult to adjust its existing practices and product range to fit high density mixed use long term PDL requirements and thus set up a specialised subsidiary to exploit a specific market niche: the inner city high margin markets of London and Manchester. The activities of the subsidiary grew significantly in the few years following its establishment but eventually it was deemed to be exposing Wimpey to risks that the company did not want to assume. At the same time other Wimpey subsidiaries were also beginning to develop PDL sites but part of their effort was focused on lower density PDL whereas the company's capacity to develop high density developments was also limited by the traditional housebuilding construction methods. Thus it appears that George Wimpey had some difficulty in realigning itself to the new business environment it was faced with, its financial position deteriorated and eventually the company merged with another top-10 housebuilding firm in order to avoid bankruptcy.

The Berkeley Group on the other hand transformed itself from a small housebuilder of exclusive suburban detached housing to a ‘regenerator’ at the forefront of mixed use long term PDL redevelopment. It tested the market early on in the 1990s and strategically aligned itself with government policy. It managed market uncertainty through a flexible approach towards development which is based on flexibility towards the planning and design of the product and relying on the incorporation of market feedback into the development process.

The company does not tackle market volatility by diversifying in terms of geography and market segments. Instead it specialises in upmarket, high margin, mixed use developments in relatively few selected markets and prominent locations. During periods of housing market downturns the commercial and affordable housing activities are partly compensating for the decline in profits from speculative housebuilding whereas significant amounts of capital and channelled towards land buying. There are no geographical or other boundaries between company subsidiaries but in any case the Group is only active in London and the South East of England and almost exclusively engaged in PDL development. The outcome of this strategic approach to development is that the company has grown at a dramatic pace during the boom years and has built a dominant position in the London and South East market which allowed it to stay profitable during the downturn. Its profit margins are steadily high in part due to its capacity to exploit strategic land opportunities.

A major element of any housebuilder's strategy is the preoccupation with market volatility and the housebuilder's inability to predict which way the market will turn next. The switch from non-PDL to PDL caused both housebuilders to restructure their landbanks, a key element of their competitive strategy and their profitability. This reorganisation is a very difficult thing to achieve in the short term and it incurs substantial costs to housebuilders. Other than that, the two housebuilders had to rethink the way they manage the development process. The traditional sequential approach was challenged by approaches stressing flexibility, mixed uses, phasing and contemporality, in what can be termed the ‘development management’ approach. As Ball (1983) has eloquently discussed, this volatility forces most housebuilding firms to adopt production methods that would allow them to stop production and exit the market very quickly if the market takes a downwards trend and at the same time would allow them to speed up the production cycle if the market is booming. In a business environment were the final product is a single-use housing development, a sensible housebuilding firm would subcontract their labour force, maintain minimal investment on capital assets and use a landbank to regulate the flow of land in the development pipeline. The emphasis is on speed and on the ability to stop production and stay on the sidelines during a market crash, then get back in when things get better. The additional effect of speeding the cycle is the improvement on the Return on Capital Employed (ROCE), a key indicator used by investors. This preoccupation with market volatility is ever present but the policy requirements during the 2000s to engage in long term projects and produce mixed-use multifunctional urban environments on PDL challenged the traditional approaches designed to tackle that volatility.

The shift to PDL was both an opportunity and a threat. A threat because mixed use PDL schemes are more complicated to plan, design, build and manage than a single use scheme on non-PDL. This means that a prolonged and thorough engagement of the developer with the development is necessary. At the same time mixed use schemes allow simultaneous access to more than one property markets. In that sense a mixed use scheme can be seen as a portfolio diversification opportunity, where mix of uses is adjusted in order to mitigate market risks.

Berkeley manages these uncertainties by approaching development flexibly. A long term engagement with development of a site is seen as normal from a company that calls itself a ‘Regenerator’. Market volatility is therefore managed or even exploited through diversification and phasing. The type of uses and the composition of the development (types of apartments built, square metres of each use) is under almost constant adjustment. This adjustment is based on what the multiskilled teams managing the project (and the development manager leading them) think that the market wants at the time when construction in each phase of the development begins. This flexibility requires an equally continuous renegotiation of the planning permission which in turn requires a good working relation with the Local Planning Authority. At the project level, the emphasis on flexibility and, to some extent, on collaboration contribute to the firm's competitive advantage.

Focusing on the construction element of housing development is only part of the picture. Planning policy promotes higher densities albeit for reasons like liveability, land conservation, energy efficiency and public transport viability. This means that in traditionally built housing developments on urban PDL the need to maintain or increase margins puts pressure on ‘affordable housing’ provision and/or quality and unit size standards. Higher densities mean more capital intensive development, in fact the construction costs of high density are so much higher that whereas in non-PDL housebuilding the cost of land comprises up to 40% of the total cost, in PDL housebuilding this is closer to 10%. Indeed, one of the biggest early achievements of Taylor Wimpey was that they managed to stabilise selling prices but brought purchase price per plot at £30,000 compared to £45,000 that George Wimpey was paying per plot just before the merger.

Interestingly, for buildings above six to seven storeys there needs to be a ‘transition’ into different technologies and materials (structural concrete, curtain walling etc.) that are more expensive per square metre and their use makes much more financial sense when applied to high-rise blocks. This technological transition requires a big investment in know-how and a significant restructuring of the supply chain of the producers. Faced with this reality, one of the housebuilders looked into adopted the new technological paradigm and abandoned some key aspects of the old way of housebuilding altogether without shedding off other important elements like strategic landbanking.

This result accords with the expectations of the theoretical insights explored in section 2 and is a remarkable conclusion since Berkeley took a ‘risky’ approach if seen under the lens of conventional housebuilding. It radically transformed itself from a small suburban high end developer to a major housebuilder engaging in long term, complex mixed use schemes. However, the Berkeley approach turned out to be a well calculated and orchestrated exercise in capacity building that pre-empted the changes in the business environment.

For the housebuilding sector as a whole it took four to five years to respond in the changes in government policy at a scale big enough to make a difference. In Fig. 3.1 it is evident that the average density of dwellings in new developments suddenly leaped between 2001 and 2003 and then almost stabilised from 2006 onward regardless of the market conditions. If one accepts that higher density development is initself more sustainable but also a proxy for potentially more sustainable built environments this leap in densities post-2002 indicates a structural shift and in effect demonstrates that the aim of the government to steer the housebuilding sector towards what it understood as more ‘sustainable’ forms of development was relatively successful. Indeed, other indicators, like land consumption also point to the same direction (Karadimitriou, 2005). Thus one could plausibly argue that until 2007 the housebuilding industry was indeed satisfying the government agenda to an extent, notwithstanding the perennial problem of low overall production. The analysis in this paper is also an interesting insight into how housebuilders may have achieved this by changing their ‘ways of doing things’ and thus the configurations of products they offered.

Since 2007 however, there seems to be a new trend emerging, one that may actually become even more puzzling and more difficult to label as ‘sustainable’ as the new government agenda begins to affect housebuilding. This new trend combines a substantial drop in overall housing production, average densities of new developments remaining stable at pre-2007 highs and a drop in the proportion of new dwellings that are flats. This may well mean that the UK housebuilding sector is entering a very polarised era where production will comprise on the one hand very high density developments in urban areas (mainly London, mostly Berkeley) and on the other hand low density multi-bedroom houses in more rural areas. Would that be a sustainable outcome, especially in an era of persistently high dwelling prices, tight mortgage credit and static incomes?

In terms of future research, other than the questions above, this paper poses the question whether the practices of the Berkeley Group were indeed an industry-wide trend and if that is the case, how successful any other companies were and why they now seem to be abandoning that know-how. There are indications that Laing and McAlpine were pursuing strategies similar to Berkeley's until they were bought by Wimpey but other smaller or bigger developers might also have things to offer in that respect. By the same token it would be very interesting to examine how housebuilders specialising in greenfield development in rural or non-metropolitan areas are currently responding to the introduction of the Localism Act and the NPPF.

Community participation will in all likelihood be a key factor affecting future housebuilder practices, in a context of combined localism and NIMBYism. It is worth pointing out here that the ‘pre-application consultation’ requirements which the Localism Act introduced in 2011 were in fact a key element of the approach that Berkeley (and other developers) were following. It is a clear indication of how policy and business practice can and often do evolve together, feeding into each other. In the ‘development management’ approach there is no clear idea from the outset about what the development will comprise at the end. Such certainty does not exist throughout the process. This however means that the LPA and local communities are facing difficulties in negotiating planning obligations whereas tenants who move in at the early stages may find themselves in front of unexpected surprises, for better or for worse. The urban environments that are built today as a result of long terms mixed use PDL schemes will reflect what the market wants at each phase of their development but might have a problem in actually reflecting what the wider needs and aspirations of present and future residents might be.

## Figures and Tables

**Picture 1:**
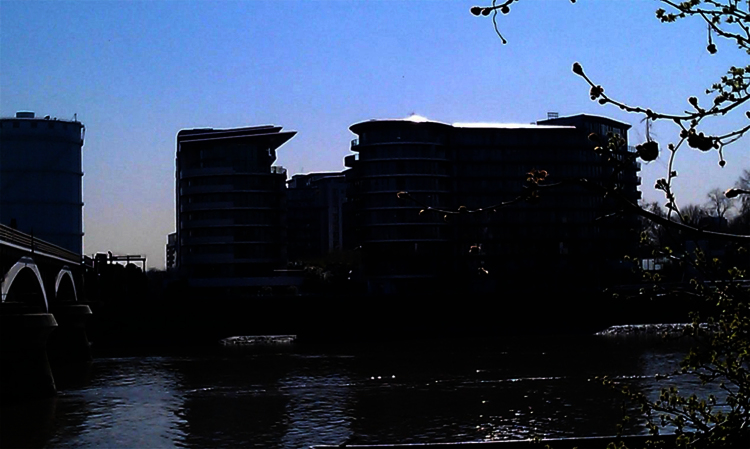
Northern view of Chelsea Bridge Wharf.

*Source*: Author.

**Picture 2:**
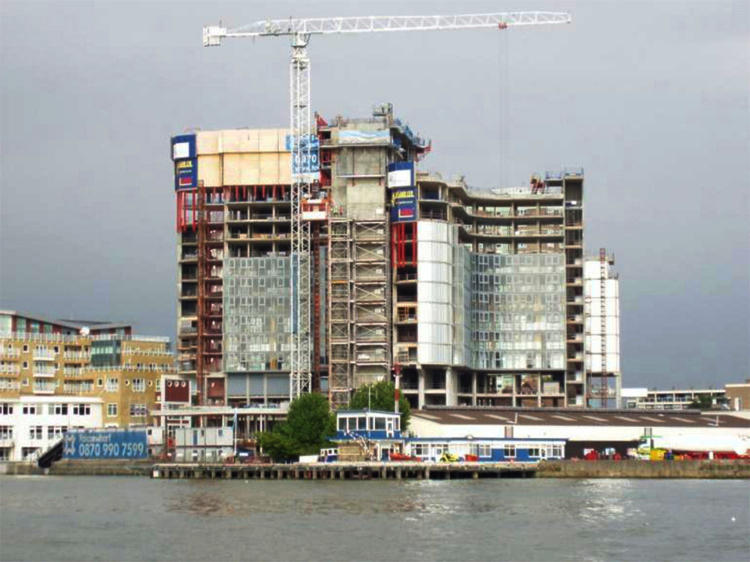
Falcon Wharf under construction.

*Source*: Author.

**Table 1:** Trends in total dwelling production by density and land type during 1994–2003.

	High density 1999–2003	High density 1994–1998	Trend	Medium density 1999–2003	Medium density 1994–1998	Trend	Low density 1999–2003	Low density 1994–1998	Trend
Non-PDL	6%	5%	UP	18%	16%	UP	76%	79%	DOWN
PDL	22%	17%	UP	24%	25%	DOWN	54%	58%	DOWN

**Table 2:** The six land-density options open to housebuilders.

	High density (>80 dw/ha)	Medium density (30–80 dw/ha)	Low density (<30 dw/ha)
Type of land			
Non-PDL	Similar to high rise layouts tried in the 1950s and 1960s. The planning system and local NIMBYism make it unlikely that such developments could go ahead easily	More likely than high density non-PDL to be accepted by LPAs and local communities. Traditional layouts and housebuilding construction methods can still be used	Traditional suburban housing developments, promoted by planning policy until 1995 and perhaps more acceptable again insofar as planning policy is concerned. Generally more acceptable by suburban and rural local communities

PDL	Financially profitable at high enough densities. Construction methods different to traditional housebuilding (concrete, curtain walling etc.). Changing social norms and policy discourses make it more acceptable. ‘Urban chic’ marketing appealing to certain retail customers whereas the format is suitable for wholesale purchases by investors	More likely to be accepted by LPAs and local communities. Often more efficient and profitable for housebuilders to switch to different methods and higher densities	An attempt to create the same configuration of space as above using a less advantageous type of land (higher servicing costs, more expensive, etc.). Difficult to capitalise on the ‘rural idyll’ since PDL is rarely rural

**Table 3:** The change in the use mix following the initial planning permission.

	Flats (aff/ble)	Density (d.p.h.)	1-Bed	2-Bed	3-Bed	Hotel	Restaurant and bar (m^2^)	Health club (m^2^)	Retail (m^2^)	Office (m^2^)
Original Applic. 2000–2001	608 (152)	173	197	382	29	235 beds	2800	3500	370	8490
Situation 2004	723	206	n.av.	n.av.	n.av.	438 beds	Smaller but m^2^ n.av.	3500	370	Smaller but m^2^ n.av.
Situation 2010	∼880	∼257	n.av	n.av	n.av	213 beds	∼1500	∼800	∼3400	∼4000

**Table 4:** The change in the use mix following planning permission in Falcon Wharf.

	Flats (aff/ble)	Live-work (units)	Density (dw/ha)	Leisure, etc. (m^2^)	1-Bed	2-Bed	3-Bed	Other dwellings	Hotel	Office (m^2^)
Harbour Land application 2000	149 (35)	8	300	668 restaurant	65	44	10	24 studios, 6 penth.	87 beds	4171 (alt.)
GWC application 2002 (perm. granted 2005)	145 (21 + 14 off site)	–	292	746 restaurant	87	47	10	1 penth.	–	3048
Situation 2010	143 (21 +14 off site)	–	288	446 restaurant, 744 spa and gum	87	44	11	1 enlarged penth.	60 beds	–

**Table 5:**
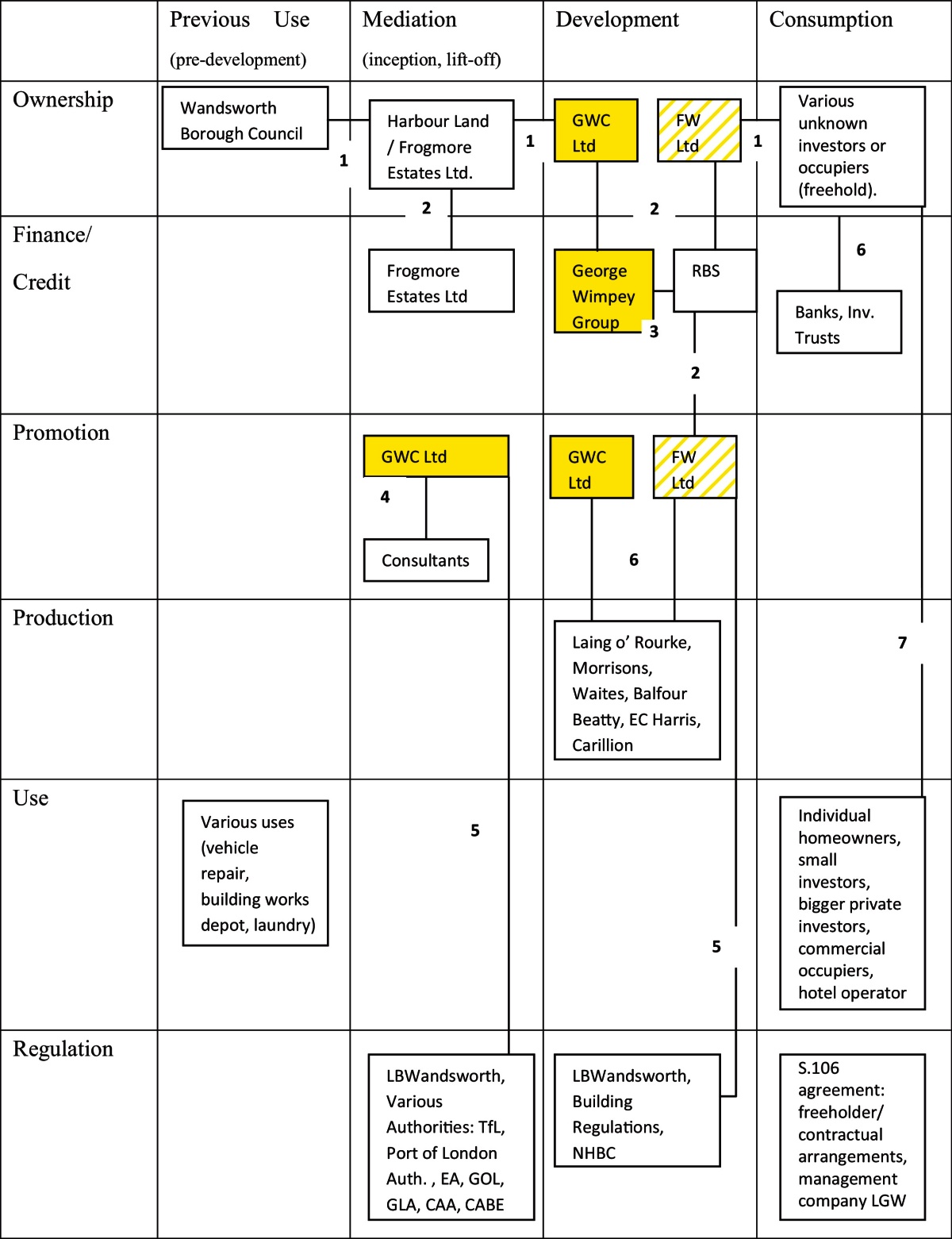
Functions, phases and interactions in the development process.

## Footnotes

1 The examination of the ‘interaction dynamics’ of selection however are a much more complex issue. See Dosi (2000, p. 25).

2 This behaviour is hardly ‘profit maximising’ in the sense of a “…a global, faultless, once-and-for-all optimisation over a given choice set comprising all objectively available alternatives.” (Nelson & Winter, 1982, p. 31).

3 In the case of the Berkeley Group these profits include profits from activities outside the UK which are minimal as well as non housebuilding activities.

4 Double tracking’ means that the developer submitted the same application twice simultaneously. In that case, if one application does not get planning permission and an appeal is launched there is still one application to be determined by the LPA which means that negotiations can continue while the appeal is pending.
